# Nanofibrous Porous Organic Polymers and Their Derivatives: From Synthesis to Applications

**DOI:** 10.1002/advs.202400626

**Published:** 2024-03-12

**Authors:** Chen Yang, Kexiang Wang, Wei Lyu, He Liu, Jiaqiang Li, Yue Wang, Ruyu Jiang, Jiayin Yuan, Yaozu Liao

**Affiliations:** ^1^ State Key Laboratory for Modification of Chemical Fibers and Polymer Materials, College of Materials Science and Engineering Donghua University Shanghai 201620 China; ^2^ Department of Materials and Environmental Chemistry Stockholm University Stockholm 10691 Sweden

**Keywords:** applications, bulk synthesis, electrospinning, nanofibers, porous organic polymers

## Abstract

Engineering porous organic polymers (POPs) into 1D morphology holds significant promise for diverse applications due to their exceptional processability and increased surface contact for enhanced interactions with guest molecules. This article reviews the latest developments in nanofibrous POPs and their derivatives, encompassing porous organic polymer nanofibers, their composites, and POPs‐derived carbon nanofibers. The review delves into the design and fabrication strategies, elucidates the formation mechanisms, explores their functional attributes, and highlights promising applications. The first section systematically outlines two primary fabrication approaches of nanofibrous POPs, i.e., direct bulk synthesis and electrospinning technology. Both routes are discussed and compared in terms of template utilization and post‐treatments. Next, performance of nanofibrous POPs and their derivatives are reviewed for applications including water treatment, water/oil separation, gas adsorption, energy storage, heterogeneous catalysis, microwave absorption, and biomedical systems. Finally, highlighting existent challenges and offering future prospects of nanofibrous POPs and their derivatives are concluded.

## Graphical abstract

In this review, the authors provide a comprehensive overview of porous organic polymers （POPs）‐based nanofibers and their derivatives. The coverage includes synthesis, preparation methodologies, and emerging applications, offering the first exhaustive examination of various nanofibrous POPs, their composite materials, and derived carbon materials. Additionally, the authors delve into the diverse applications, challenges, and future perspectives of these nanofibers.

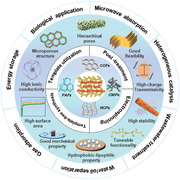

## 1 Introduction

Recently there has been a notable surge in the exploration of porous materials, encompassing zeolites, activated carbon, mesoporous silica, metal‐organic frameworks, porous organic polymers (POPs) and more. These materials, characterized by controllable voids from molecular to nanoscopic scales, hold immense promise as problem‐solvers across diverse fields, spanning from separation, catalysis, adsorption, energy storage, to ion exchange.^[^ ^1^, ^2^, ^3^, ^4^, ^5^, ^6^, ^7^, ^8^ ^]^ Among them, POPs stand out as an innovative category, composed of abundant and lightweight constituent elements such as carbon, hydrogen, oxygen, and others. These elements covalently bond to create POPs with advantageous attributes, e.g., low density, customizable pore structures and porosities, substantial surface areas, and robust thermal and chemical stability.^[^ ^9^, ^10^, ^11^, ^12^, ^13^, ^14^, ^15^ ^]^ Notably, the wealth of organic building blocks within POPs provides unparalleled opportunities for precise molecular structure engineering to fine‐tune their structures and functions.^[^ ^16^, ^17^, ^18^ ^]^ POPs have showcased substantial potential in a wide array of applications, encompassing adsorption,^[^ ^19^ ^]^ separation,^[^ ^20^, ^21^ ^]^ electrochemical energy generation and storage,^[^ ^22^ ^]^ renewable energy conversion,^[^ ^23^, ^24^ ^]^ biomedical applications, and nanocarrier systems.^[^ ^25^, ^26^ ^]^

Broadly POPs can be categorized into two groups, i.e., amorphous and crystalline POPs. The former includes hypercrosslinked polymers (HCPs),^[^ ^27^, ^28^, ^29^, ^30^ ^]^ porous aromatic frameworks (PAFs),^[^ ^31^, ^32^, ^33^, ^34^, ^35^ ^]^ conjugated microporous polymers (CMPs),^[^ ^36^, ^37^, ^38^, ^39^, ^40^, ^41^, ^42^ ^]^ and polymers of intrinsic microporosity (PIMs).^[^ ^43^, ^44^, ^45^, ^46^ ^]^ The latter is notably exemplified by covalent organic frameworks (COFs),^[^ ^47^, ^48^, ^49^, ^50^, ^51^, ^52^, ^53^ ^]^ which exhibit periodic, ordered, and extended conjugated skeletons. To note, there are also some other amorphous POPs that typically arise from C‐C coupling reactions via traditional solvothermal synthesis methods. These materials, often existing as insoluble bulk POPs or (nano)particles with rigid crosslinked frameworks, can pose challenges due to reduced exposure of active sites and limited processability, which hinder their practical use.^[^ ^54^, ^55^ ^]^

Recent research has been actively focused on the precise construction of POPs with controlled morphologies, such as nanosheets, nanoparticles, nanovesicles, and nanofibers. These well‐defined microstructures offer enhanced suitability for task‐specific applications.^[^ ^56^, ^57^, ^58^, ^59^, ^60^ ^]^ This review particularly emphasizes the 1D nanofibrous morphology, as it, similar to 0D or 2D materials, retains the intrinsic advantages of nanoscale effects that are well‐preserved for macroscopic operation. 1D shape offers unique benefits over 3D bulk materials, including ease of processing for practical use, lightweight characteristics, tuneable surface functionality, good surface contact and reduced diffusion resistance to its interior.^[^ ^61^, ^62^, ^63^, ^64^ ^]^ No wonder that extensive efforts have been dedicated to shaping POPs into nanofibers, enhancing their utility in target systems.^[^ ^65^, ^66^, ^67^ ^]^

Research aiming at producing POPs nanofibers (POP NFs) can be broadly categorized into two approaches: direct bulk synthesis, involving hard‐template, soft‐template, and template‐free methods, and electrospinning‐aided synthesis. The formation mechanisms of POP NFs with varied chemical composition and morphologies have been summarized and comparably viewed in‐depth, where key experiment factors such as reactant concentration, monomer type, solubility, mechanical agitation, and reaction time have been systematically discussed. As a step forward, the review scope is extended to combination of template techniques with POPs for generating POP‐based nanofibrous composites (POP NFCOMs) with help of various binding forces, including covalent and non‐covalent interactions. In the electrospinning method for producing nanofibrous POPs and their derivatives, the roles of reactant concentration and solvent composition have been explored.

**Figure 1:**
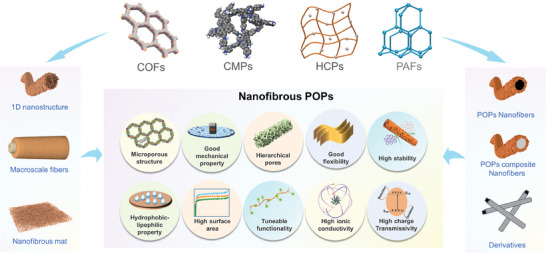
Schematic of the classifications and key property profiles of nanofibrous POPs and their derivatives.

The 1D‐derived attributes have significantly bolstered the use of POPs in various domains, such as adsorption, catalysis, wastewater treatment,^[^ ^68^, ^69^, ^70^ ^]^ separation,^[^ ^71^, ^72^ ^]^ energy storage,^[^ ^73^, ^74^, ^75^ ^]^ microwave absorption,^[^ ^76^ ^]^ sensors,^[77]^ biomedical systems,^[^ ^26^ ^]^ and more. Surprisingly, despite the rapid development and promising future perspective of nanofibrous POPs and their derivatives, comprehensive reviews on their synthesis, processing, and applications have been conspicuously absent. This knowledge gap serves as the motivation for composing this review, which comprehensively covers nanofibers derived from COFs, CMPs, HCPs, PAFs, their composites, and carbon derivatives (**Figure** **1**). Notably, the contribution excludes discussions on PIMs fibers, which was reviewed by Bekir et al. recently.^[^ ^78^ ^]^ Our review aims to provide researchers with an overview and in‐depth understanding of the state‐of‐the‐art developments in nanofibrous POPs and their derivatives, and valuable insights and perspective into their design principals and potential use.

## 2 Preparation of Nanofibrous POPs and their Derivatives

### 2.1 Bulk Synthesis

**Figure 2:**
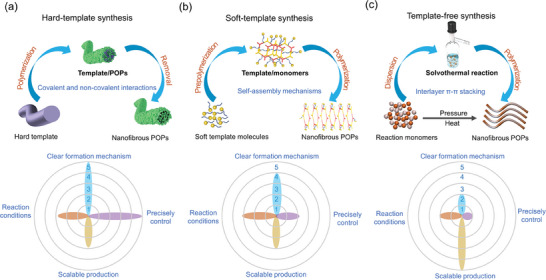
The synthesis strategies and the corresponding *pros* and *cons* of a) hard‐template, b) soft‐template, and c) template‐free synthesis.

Bulk POPs, characterized by irregular shapes and morphologies, are produced through the classic polymerization procedure. This occurs due to the inherently fast reaction kinetics of building blocks, which leads to the formation of a large number of nuclei upon contact of building blocks with each other. As a result, irregular morphologies are expected as these crystals tend to ripen in an anisotropic manner.^[^ ^79^, ^80^, ^81^ ^]^ By controlling the synthetic conditions, it is possible to shape them into well‐defined nanofibers.^[^ ^82^, ^83^, ^84^ ^]^ The bulk synthesis of POP NFs can be accomplished through three methods: hard‐template, soft‐template, and template‐free synthesis (**Figure** **2**), which are detailed below.

#### 2.1.1 Hard‐Template Synthesis

The hard‐template method refers to a synthetic approach that utilizes a specific substance as a spatial filler, and the subsequent removal of this substance results in the corresponding nanofibrous structure. This method is currently one of the most commonly employed and well‐established approaches for preparing POPs with specific morphologies. Therefore, the hard‐template synthesis is effective for producing POP NFs with precise length and diameter via the employment of well‐defined hard templates and polymerization procedures. Notably, the post‐synthetic removal of templates may undermine the as‐formed nanostructures. Unsurprisingly, without removing the template, POP NFCOMs are usually the default products. Various fibrous substrates have been reported as templates, including silica filaments,^[^ ^85^ ^]^ ITO glass,^[^ ^67^ ^]^ short polymer fibers,^[^ ^86^ ^]^ syndiotactic polystyrene aerogels,^[^ ^87^ ^]^ metal wires,^[^ ^88^, ^89^ ^]^ nylon nanofiber membranes,^[^ ^90^ ^]^ carbon nanofibers (CNFs),^[^ ^91^, ^92^ ^]^ carbon nanotubes (CNTs),^[^ ^73^, ^93^, ^94^ ^]^ and natural biofibers.^[^ ^57^ ^]^ Among these, CNFs, CNTs, stainless steel wires, and biofibers are the most popular choices due to their excellent conductivity, mechanical strength, or biodegradability.

**Figure 3:**
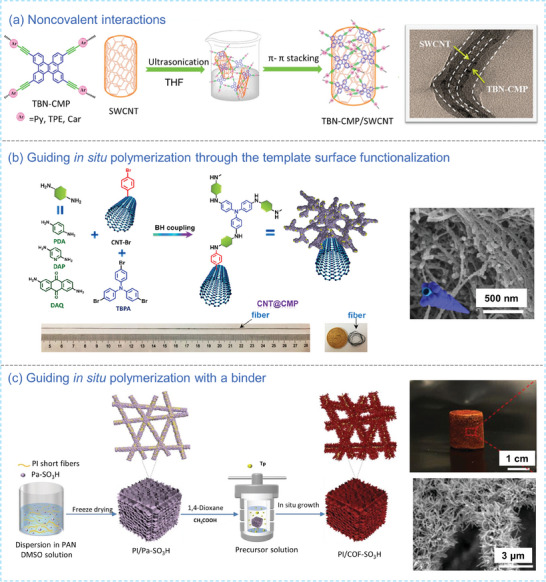
a) Schematic of the preparation procedure and SEM image of TBN‐CMP/SWCNT nanofibers through the introduction of noncovalent interactions.^[^ ^96^ ^]^ Copyright 2021, American Chemical Society. b) Synthesis schematic and SEM image of CNF@CMP nanofibers achieved by functionalizing the template surface.^[^ ^98^ ^]^ Copyright 2021, American Chemical Society. c) Synthesis schematic, digital photograph and SEM image of PI/COF‐SO_3_H through the introduction of a PAN binder.^[^ ^86^ ^]^ Copyright 2023, Wiley‐VCH.

POP NFCOMs can be prepared by introducing noncovalent interactions between the hard template and POPs, or by functionalizing the template surface with covalent bonds to guide the in situ of POPs via polymerization.^[^ ^95^ ^]^ For instance, Samy et al.^[^ ^96^ ^]^ prepared nanofibrous nanocomposites by blending and attaching tetrabenzonaphthalene (TBN)‐based CMPs onto single‐walled CNT (SWCNT) via π‐π interactions under sonication treatment (**Figure** **3**a). Li et al.^[^ ^97^ ^]^ aminated cotton fibers through a silylation reaction with (3‐aminopropyl)trimethoxysilane and then in situ grafted 2D imine‐linked COFs on the fiber surface via a Schiff base reaction, resulting in dimensionally controllable nanofibers with excellent mechanical properties and stability. Lyu et al.^[^ ^98^ ^]^ covalently bromo‐functionalized CNFs, which reacted with polytriphenylamine (PTPA) in Buchwald‐Hartwig (B‐H) coupling reaction to obtain PTPA‐grafted CNF fibers (PTPA@CNFs) (Figure 3b). A core‐shell fibrous composite with a high surface area was also achieved using spirobifluorene‐based CMP via a similar surface‐grafting modification method.^[^ ^99^ ^]^ The incorporation of a polymer binder to enhance adhesion between the POPs and the template is another commonly employed strategy in crafting POP NFCOMs. Agarwal et al.^[^ ^86^ ^]^ employed polyacrylonitrile (PAN) as a binder to guide the polymerization of 1,3,5‐triformylphloroglucinol (Tp) and 2,5‐diaminobenzenesulfonic acid on polyimide (PI) short fiber sponge via a Schiff base reaction (Figure 3c). Within this reaction, PAN secured the structural integrity of the sponge and served as an active site for the in situ polymerization of COF‐SO_3_H. Owing to the strong π‐π interactions, 3D crystalline COF sponges (PI/COF‐SO_3_H) interconnected by nanowires with high mechanical property was finally afforded.

**Figure 4:**
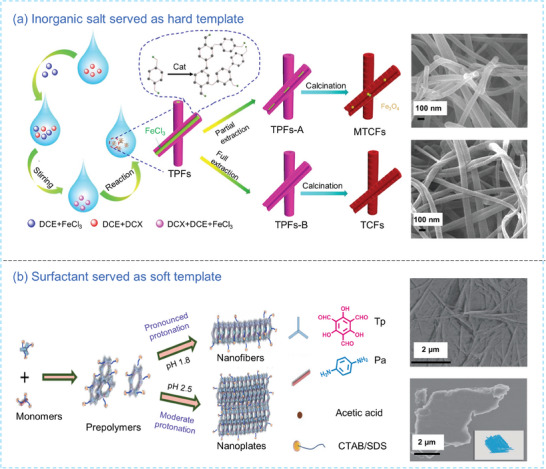
a) Synthetic scheme and SEM images of TPFs and magnetic TCFs (MTCFs) using FeCl_3_ as the hard template.^[^ ^100^ ^]^ Copyright 2019, Wiley‐VCH. b) Synthetic scheme and SEM images of triformylphloroglucinol phenylenediamine (TpPa) using surfactants (CTAB and SDS) as the soft template.^[^ ^102^ ^]^ Copyright 2022, Springer Nature.

Another class of hard templates is Lewis acid catalysts. For instance, crystallized FeCl_3_ in a needle shape has been adopted as a 1D template in hypercrosslinking polymerization of α,α'‐dichloro‐p‐xylene (DCX) via Friedel‐Crafts alkylation at a suitable temperature exceeding the boiling point of the soluble oil solvent.^[^ ^100^ ^]^ In the polymerization process, the catalyst (FeCl_3_) in a needle form was loaded, and the polymer grew as a thin sheath externally onto the nanofiber. Further removal of the FeCl_3_ template by solvent extraction results in tubular polymer nanofibers (TPFs), which are carbonized into tubular carbon nanofibers (TCFs) maintaining the similar tubular morphology (**Figure** **4**a). In another attempt, Bhaumik and co‐workers^[^ ^101^ ^]^ used FeCl_3_ to catalyze the self‐polymerization of triphenylamine, which grew along the c‐axis along the 001 direction to form porous polymer nanofibers.

POP NFCOMs[Supplementary-material advs7693-supitem-0001] obtained through noncovalent interactions as binding forces are expected to exhibit good stability and compactness. Unfortunately, available templates for noncovalent interactions are limited. Conversely, nanofibers obtained through covalent interactions between POPs and templates demonstrate the best stability and dispersibility. More importantly, the loading amount of POPs nanoparticles (POP NPs) on the surface of templates can be controlled. However, it's important to note that the hard template needs to be removed by heat treatment or strong acid and alkali after synthesis, which may undermine the structural integrity of the formed nanostructures. This is a significant drawback of the hard‐template method. Additionally, the hard template method faces challenges when it comes to large‐scale production of POP NFs.

#### 2.1.2 Soft‐Template Synthesis

A comparably simple, mild reaction condition, widely applicable, and powerful method for preparing POP NFs is to use soft templates. This method is based on self‐assembly mechanisms involving *van der Waals* forces, hydrogen bonding, electrostatic interactions, π‐π stacking interactions, and/or metal coordination as driving forces. Different from the hard‐template method, the soft‐template method incorporates soluble or removable organic polymers as a template, which can be easily removed after the completion of the reaction. The main challenge in this method is to precisely control the morphology, diameter, and orientation of soft templates. In general, the molecules used to build up the soft template, acting as stabilizers, form micelles or nanofibers to aggregate the reactive monomers around them during the initial stages of polymerization. These templates then guide the desirable growth of polymers in a nanofibrous form under appropriate reaction conditions.

Surfactants are among the most commonly used soft templates, as they are cheap and create thermodynamically stable micelles in solution, which effectively control the formation of POP NFs. For instance, Yang et al.^[^ ^102^ ^]^ employed hexadecyltrimethylammonium bromide (CTAB) and sodium dodecyl sulfate (SDS) surfactants as stabilizers for monomers and prepolymers to generate micelles in an acid‐catalyzed Schiff base polymerization process (Figure 4b). These co‐formed micelles act as a precise medium, regulating the formation of COF nanofibers. This morphological regulation involves the slow‐down of the reaction kinetics and the curbing of the ripening process of the prepolymer, especially at a pH of 1.8. In this 1D confinement environment, the prepolymers grew into well‐defined nanofibers and further evolved into long fibers via noncovalent interaction between the COF interlayers as the driving force.

#### 2.1.3 Template‐Free Synthesis

The template‐free method is characterized by its simplicity and rapidity, eliminating the need for template removal. The resulting POPs nanofibers exhibiting high purity and yield, enabling scalable production. Template‐free synthesis is well‐suited for scalable production of POPs with nanofibrous morphology through one‐step polymerization. This approach relies on the careful manipulation of synthetic conditions, which govern the thermodynamics and kinetics of the solvothermal reaction. The rational optimization of the reaction parameters encompasses a range of factors, including the choice of monomers, catalysts, additives, reaction time, temperature, and solvents. The formation of 1D nanofibers primarily arises from the monomer‐induced interlayer π‐π stacking process, which directs growth of POPs to mature along the Z‐direction. Recently, solvothermal reactions, such as the Schiff base reaction,^[^ ^67^, ^103^, ^104^, ^105^ ^]^ Knoevenagel condensation reaction,^[^ ^106^, ^107^ ^]^ Sonogashira‐Hagihara cross‐coupling condensation reaction,^[^ ^108^, ^109^ ^]^ and oxidative homocoupling reaction,^[^ ^110^ ^]^ have been explored in the template‐free methods for synthesizing POP‐NFs.

**Figure 5:**
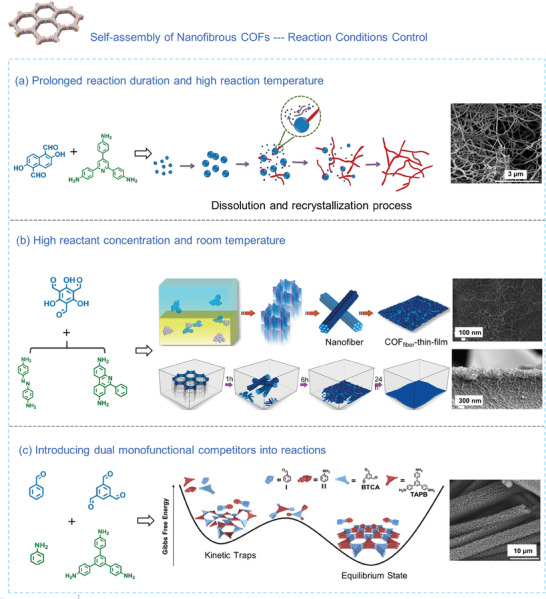
a) Synthetic scheme illustrating the dissolution‐recrystallization process of nanofibrous COF along with its SEM image.^[^ ^111^ ^]^ Copyright 2021, American Chemical Society. b) Schematic of the self‐assembly process and SEM images of nanofibrous COF thin film obtained under prolonged reaction time and high reactant concentrations.^[^ ^112^ ^]^ Copyright 2022, Elsevier. c) Schematic of the Gibbs free energies during the self‐assembly process in the reaction system involving two monofunctional competitors, accompanied by the corresponding SEM image of COF hollow fibers.^[^ ^113^ ^]^ Copyright 2019, Elsevier.

The Schiff base reaction is well‐investigated for preparing COFs. Numerous studies have demonstrated that COF nanoparticles can be induced to disassemble and then reassemble into 1D nanofibrous structures by judiciously controlling reaction parameters, e.g., reactant concentration, solvent composition, reaction time, and temperature. The driving force behind this transformation lies in the reversible dynamics of imine bonds, which facilitate a unique dissolution‐recrystallization process for COFs (**Figure** **5**a). During this procedure, extended reaction duration and elevated temperatures are frequently necessary to boost the crystallinity of COF and induce a transformation in morphology (180 °C, 24 h). Irregular nanoparticles initially form through homogeneous nucleation, after which they aggregate into uniformly sized microspheres. These microspheres then undergo partial dissolution, followed by further reaction and recrystallization into smaller nucleation sites of higher energy. Eventually, this process leads to the formation of well‐defined crystalline COF nanofibers along the 1D direction.^[^ ^111^ ^]^ By increasing the concentration of reactants can also achieve nanofibrous COFs. Banerjee's team introduced Tp and two distinct NH_2_‐gruop monomers in an open system at high reactant concentrations at room temperature. Through an extended reaction of self‐assembly, nanofibrous COF thin films were produced. Conversely, when utilizing a closed system at low reactant concentrations, only nanospheres COF‐derived thin films were obtained (Figure 5b).^[^ ^112^ ^]^

In 2019, a “reversible polycondensation‐termination” (RPT) method was used to fabricate highly crystalline COFs. This method involved two monofunctional competitors into the reaction system (Figure 5c). In a typical Schiff base system, monofunctional amine (I) and aldehyde (II) were introduced as competitive building blocks into 0.10–7.50 mm of 1,3,5‐tris(4‐aminophenyl)benzene (TAPB) and 1,3,5‐benzenetricarbaldehyde. This addition aimed to strengthen reversible associations within the RPT method. The heightened reversibility was found to lower the Gibbs free energy of COFs, effectively releasing the reaction system from kinetic constraints and allowing it to reach an equilibrium state. Consequently, the RPT method demonstrated effective control over the preparation of COFs with well‐defined morphologies. This control was achieved by adjusting various reaction parameters such as reaction time, solvent composition, and reactant concentration, but notably simultaneous addition of monofunctional amine and aldehyde was crucial for optimal results. Specifically, when appropriate solvent types, solvent ratios, reaction temperatures, and times were employed, along with an initial reactant concentration exceeding 0.25 mm, COF hollow fibers were successfully synthesized.^[^ ^113^ ^]^

**Figure 6:**
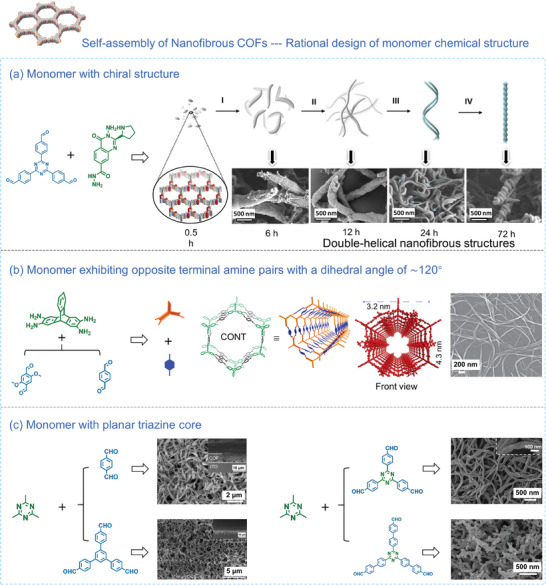
a) Schematic of the morphological evolution process leading to double‐helical nanofibrous structures through the selection of monomers with a chiral structure.^[^ ^105^ ^]^ Copyright 2023, Wiley‐VCH. b) Self‐assembly process schematic of hollow nanofibrous COF utilizing TAT as the core.^[^ ^114^ ^]^ Copyright 2022, Springer Nature. c) Synthetic pathway for nanofibrous COF formation using planar triazine monomers as the core in a Knoevenagel condensation reaction.^[^ ^106^, ^107^ ^]^ Copyright 2019, American Chemical Society and Copyright 2020, Elsevier.

The rational design of monomers with suitable chemical structure also plays a pivotal role in the template‐free self‐assembly method to form nanofibrous COFs. Recently, Liu's group^[^ ^105^ ^]^ successfully fabricated two chiral COFs (D‐PDC‐TZ COF and L‐PDC‐TZ COF) featuring distinct one‐handed double‐helical nanofibrous structures. It was accomplished by using 4,4′,4′’‐(1,3,5‐ triazine‐2,4,6‐triyl) tribenzaldehyde (TZ)and (R)‐ and (S)−3‐amino‐4‐oxo‐2‐(pyrrolidin‐2‐yl)−3,4‐dihydroquinazoline‐7‐carbohydrazide (D‐PDC and L‐PDC) as building units (**Figure** **6**a). The synthesis leveraged the non‐enantioselective generation of aminol linkages, with rod‐shaped aggregates formed at the onset of the reaction. As the reaction progressed, reactive amine and aldehyde condensed at the interface with unreacted precursors, resulting in grain boundary fusion. This process induced anisotropic morphological changes that gradually evolved into nanofibers. With prolonged reaction time, the flexible and regular nanofibers achieved sufficient length, causing adjacent nanofibers to distort and entwine, ultimately leading to the creation of a helical structure with a preferred handedness. This represents the first example of a one‐step, template‐free method involving chiral cyclic amide bonds in synthesizing helical COFs with double‐helical nanofibrous structures. Another type of 1D covalent organic nanotubes (CONTs) can be obtained by adopting tetraaminotriptycene (TAT) monomer. It possesses two diametrically opposed amine pairs with a dihedral angle of ≈120°, facilitating the formation of 1D covalent bonds. Coupling it with a linear ditopic ligand through a reversible Schiff base reaction, 1D COF hollow fibers, termed CONTs, was achieved (Figure 6b).^[^ ^114^ ^]^ Using a planar triazine monomer as the core component, a variety of olefin‐linked COFs with nanofibrous structures could also be successfully synthesized through the Knoevenagel condensation reaction.^[^ ^106^, ^107^ ^]^ These fibrous morphologies are likely the result of π‐π stacking interactions between planar triazine moieties within the COF layers, which act as the driving force to prompt the 2D COF layers to grow along a single direction, ultimately forming long fibers (Figure 6c). A similar phenomenon has been observed in the case of pyrene‐based benzimidazole‐linked nanofibers, where the self‐assembly of these nanofibers is driven by π‐π stacking interactions from the pyrene cores.^[^ ^110^ ^]^

**Figure 7:**
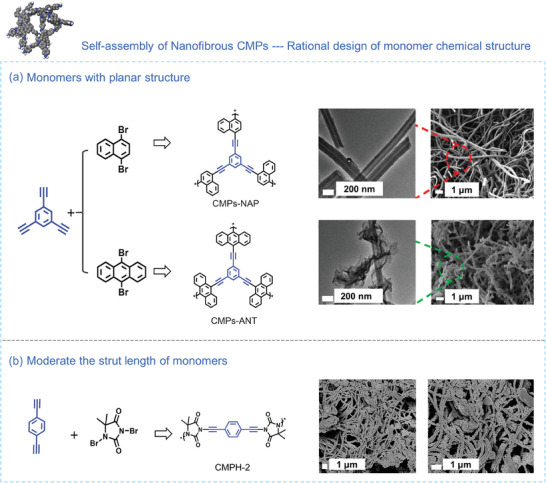
a) The synthetic pathway involving planar monomers for nanofibrous CMPs.^[^ ^109^ ^]^ Copyright 2022, Elsevier. b) The synthetic pathway for nanofibrous CMPH‐2 achieved by choosing an appropriate strut length of building blocks.^[^ ^115^ ^]^ Copyright 2018, Royal Society of Chemistry.

Through the modulation of monomer planarity, the morphology of CMPs can also be adjusted, transitioning from spherical structures to nanofibers in a one‐pot Sonogashira cross‐coupling reaction system involving bromated hydantoin and alkynyl benzene.^[^ ^109^, ^115^ ^]^ Li's group^[^ ^109^ ^]^ employed planar 1,3,5‐triethynylbenzene and bromated monomers to prepare robust and hierarchically porous CMP hollow nanofibers (CMPs‐NAP). These hollow fibers have an inner and outer diameter ranging from 60–100 nm and 120–200 nm, respectively (**Figure** **7**a). It's worth noting that side groups can sometimes impede the formation of nanofibrous structures. For instance, in the case of p[2,4,6‐(tri‐2‐thienyl)−1,3,5‐triazine (TTT)‐benzene (Ben)] CMP (pTTT‐Ben), nanofibrous structures were achieved by employing planar triazine and benzene as the core and linker, respectively, in a one‐pot polymerization reaction. However, when benzothiadiazole and dimethoxybenzene linkers were chosen, the result was random bulk particles.^[^ ^116^ ^]^ This indicates that monomers with planarity facilitate an accelerated polymer growth along the Z direction. Another determinant of the morphological characteristics in nanofibrous CMPs is the strut length of the monomers. In 2018, Li et al.^[^ ^115^ ^]^ fabricated fibrous CMPs (CMPH‐2) by adopting 1,4‐diethynylbenzene and bromated hydantoin in a Sonogashira‐Hagihara cross‐coupling condensation reaction. However, when using 1,3,5‐triethynylbenzene with varying strut lengths, spherical CMPs were obtained (Figure 7b). This variation is likely attributed to the impact of different monomer strut lengths on the conjugated system and arrangement during polymerization.

**Figure 8:**
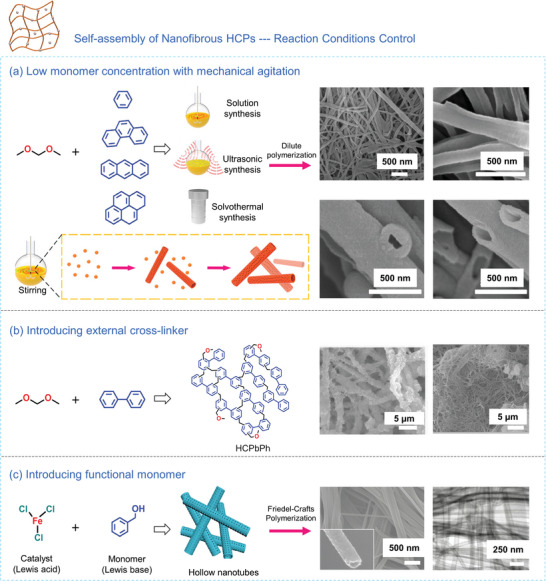
a) Synthetic scheme showing the evolution of nanofibrous HCPs obtained under low monomer concentration and mechanical agitation.^[^ ^117^ ^]^ Copyright 2017, American Chemical Society. b) Synthetic scheme showing the nanofibrous HCPs through the introduction of an external cross‐linker.^[^ ^74^ ^]^ Copyright 2021, Elsevier. c) Schematic illustrating the process of preparing hollow nanotube achieved by selecting functional monomers.^[^ ^84^ ^]^ Copyright 2019, American Chemical Society.

In the context of Friedel‐Crafts alkylation reaction, 1D HCPs can be prepared through incorporating various aromatic hydrocarbons as structural units and carefully controlling reaction parameters such as monomer concentration, reaction duration, and mechanical agitation. Jiang's team^[^ ^117^ ^]^ has pinpointed homogeneous nucleation as the primary driving force behind nanotube formation, particularly evident when utilizing a low monomer concentration (<0.1 m) or employing mechanical agitation to effectively disperse nanoparticles within the solution. Taking this a step further, the carbonization of HCP nanotubes results in the production of tubular carbon nanomaterials in a similar shape (**Figure** **8**a). Following this, Kim's group^[^ ^74^ ^]^ demonstrated that the polymer morphology is dependent on the concentration of the external cross‐linker. By employing formaldehyde dimethyl acetal (FAD) as the cross‐linker, the cross‐linking degree and the resulting nanostructure of HCPs were observed. With an increase in FAD concentration, the polymer morphology transitioned from nanospheres to nanofibers, culminating in the formation of a flower‐like nanostructure (Figure 8b). Song et al.^[^ ^84^ ^]^ proposed a growth mechanism mediated by Lewis acid‐base interactions to fabricate well‐defined HCPs. They achieved this by intelligently manipulating the structural characteristics of functional aromatic hydrocarbon monomers during the Friedel‐Crafts reaction, with the selection of functional benzyl alcohol as a monomer leading to the formation of 1D nanotubes (Figure 8c). It's worth noting that a by‐product in this reaction was believed to be pivotal in coordinating the interaction between the catalyst and monomer, ultimately driving the self‐assembly of nanotubes.

The template‐free method is characterized by its simplicity and rapidity, eliminating the need for template removal, with the resulting POPs nanofibers exhibiting high purity and yield, enabling scalable production. However, its limitations, such as imprecise control of nanofiber size, the necessity for specific monomers, and demanding reaction conditions, constrain its practical applicability. Moreover, the formation mechanism of fibrous morphologies is frequently a subject of debate, and there is currently no universally accepted design principle for the template‐free fabrication of POP‐NFs.

In summary, the three methods exhibit distinct advantages and drawbacks (Figure 2): (1) The hard‐template method enables precise modulation of nanofibrous POPs’ diameter, length, and thickness by controlling the dimensions of the hard template and polymerization time. While the formation mechanism is well‐defined, scalability is challenging. Although the reaction occurs at a moderate temperature, the intricate overall reaction conditions are necessary relatively intricate due to the requirement of heat treatment or strong acid‐base etching for hard template removal. (2) The soft‐template method can be conducted at low temperatures, and requires only solvent for template removal, making reaction conditions and scalable production more accessible compared to the hard‐template method. The formation mechanism is clear, but challenges arise in controlling the morphology, diameter, and orientation of the soft template, leading to difficulties in achieving precise control over the dimensions of nanofibrous POPs. (3) The template‐free method involves a simple, rapid one‐step polymerization process, eliminating the need for template removal and enabling scalable production. However, the current understanding of the synthesis mechanism is incomplete. In additional, achieving precise control over the morphology of nanofibrous POPs is challenging as it necessitates stringent regulation of reaction time, temperature, monomer addition, and other factors. Moreover, the majority of these processes typically require operation under high‐temperature, high‐pressure, and inert atmosphere conditions, thereby imposing rigorous demands on reaction conditions.

### 2.2 Electrospinning

**Figure 9:**
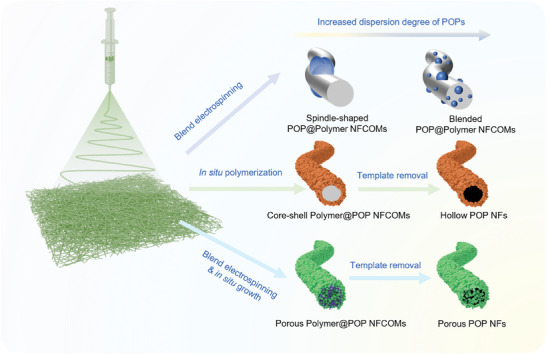
Schematic of nanofibrous POPs prepared using electrospinning technology.

Electrospinning is a technique where a viscous polymer solution overcomes surface tension by means of electrostatic forces within a high‐voltage electric field. The fluid is subsequently separated, elongated, solidified, and collected as continuous nanofibers.^[^ ^118^, ^119^, ^120^, ^121^ ^]^ Recent research has revealed that electrospinning can be employed to produce hierarchical POP NFs with additional functionalities. Broadly, three primary strategies have been utilized for generating nanofibers based on POPs through electrospinning: 1) The blend electrospinning of POP NPs with a polymer substrate, resulting in POP@Polymer NFCOMs; 2) In situ polymerization on the surface of electrospun polymer nanofibers with POPs, giving rise to Polymer@POP NFCOMs; 3) The creation of pure POP NFs mats within the realm of electrospinning technology, followed by using a substrate‐removal approach (**Figure** **9** and **Table** **1**).

**Table 1:** POP NFs fabricated via electrospinning method

Substrate materials	POPs	Methods	Average diameter [nm]	Surface area [m^2^ g^−1^]	Ref.
PAN	COF	blend electrospinning	200	17	[122]
PAN	COF‐JLU19	blend electrospinning	200–300	–	[123]
PAN	COF‐SCU1	blend electrospinning	–	–	[124]
PAN	COF‐DhaTab	blend electrospinning	200	–	[125]
PCL	CUR@COF	blend electrospinning	270	–	[126]
PES	iPAF‐6	blend electrospinning	–	–	[127]
PLA	CMP	blend electrospinning	–	–	[77]
PAN/PVP	CMP‐BT	blend electrospinning & template removal	300–500	82	[128]
PAN	COFs (TAPB‐TPA)	in situ polymerization	1000	31	[129]
PAN	COF(BT‐DG)	in situ polymerization	400	–	[130]
PAN	COF	in situ polymerization	>700	197	[131]
PVASi	Microporous organic polymer(TEDB‐NH_2_)	in situ polymerization	880	447	[132]
PVASi	POP	in situ polymerization	800	582	[133]
PANI‐PAN	PAF‐45	in situ polymerization	313	262	[134]
PVP@ Ag	CMP	in situ polymerization & template removal	300	758	[108]
PAN	TpPa COF	blend electrospinning& in situ growth& template removal	1699	1153	[71]
PS	xPNFs	stepwise crosslinking strategy	741	820	[135]
CMPES	PBF‐HNM	rigid–flexible coupling hypercross‐linking	570	579	[136]

#### 2.2.1 Blend Electrospinning Technology

POPs@Polymer NFCOMs can be directly fabricated using the blend electrospinning technique, employing pre‐synthesized POP particles that were generated under rigorous conditions. These POP NFCOMs effectively retain the porous structure and high specific surface area (SSA) of POPs while forming flexible and mechanically stable nanofiber membranes. To create a slurry for direct blend electrospinning, a mixture of POP NPs and polymers is combined in a solvent, provided that the chemical compatibility between POP NPs and the polymer in the solvent system is met. Various polymers, such as PAN, polycaprolactone (PCL), polyvinyl pyrrolidone (PVP), polyethersulfone (PES), and polylactic acid (PLA), have been chosen as the matrix for dispersing POP NPs due to their excellent stability and mechanical properties.^[^ ^137^ ^]^ These POPs@Polymer NFCOMs can take different forms, e.g., POP NPs being embedded within the nanofibers or being exposed on the nanofiber surfaces. The morphologies of these POPs@Polymer NFCOMs can be influenced by several factors, such as the loading content and the dispersion degree of POP NPs in the spinning solution, the viscosity of the spinning liquid, the input voltage, and the distance between the nozzle and collector.^[^ ^138^ ^]^

**Figure 10:**
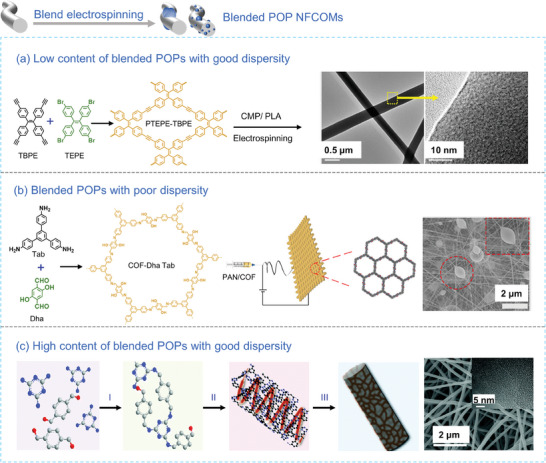
a) Synthetic scheme and TEM images of blended POP NFCOMs at a low content of blended POPs and with good dispersity.^[^ ^77^ ^]^ Copyright 2015, American Chemical Society. b) Synthetic scheme and SEM image of blended POP NFCOMs when the blended POPs exhibit poor dispersity.^[^ ^125^ ^]^ Copyright 2019, American Chemical Society. c) Synthetic scheme, SEM and TEM images of blended POP NFCOMs at a high content of blended POPs and with good dispersity.^[^ ^75^ ^]^ Copyright 2021, Royal Society of Chemistry.

Due to the inherent intermolecular π‐π stacking and hydrogen bonding within POPs, these materials typically exhibit poor dispersibility in solvents, which limits the loading capacity in composite structures. The loading content of POPs significantly influences the morphologies of the resulting composite nanofibers. At a low content of POPs, it becomes easier to obtain POPs@Polymer NFCOMs with uniform fiber diameters. For instance, Scherf et al.^[^ ^77^ ^]^ successfully fabricated a luminescent nanofibrous film composed of CMP/PLA mixtures using direct blend electrospinning technology. At a CMP content of 2.9 wt.%, the resulting CMP/PLA nanofibers exhibited a smooth surface, with the CMP component evenly distributed within the PLA matrix (**Figure** **10**a). In another study, Liu et al.^[^ ^123^ ^]^ used N, N‐dimethylformamide (DMF) as a solvent to disperse COF‐JLU19 in a PAN solution, yielding free‐standing nanofibrous membranes at varying loading ratios (0, 0.25, 0.45, and 0.65 wt.%). At a loading ratio of 0.65 wt.%, the obtained nanofibers had an average diameter of 200–300 nm. Interestingly, the blend electrospinning technique can construct spindle‐knotted composite nanofibers when the size of POP NPs exceeds the diameter of the electrospun nanofibers. For example, Zhang et al.^[^ ^125^ ^]^ engineered spindle‐knotted COF‐DhaTab/PAN nanofibers by deliberately mismatching the size between nanoparticles of COF‐DhaTab (≈200 nm) and the diameter of PAN (>200 nm), and by leveraging non‐solvent interactions (Figure 10b). The principle behind is that although the content of COF is only 3%, it cannot be dispersed into individual primary particles in the spinning solution, so a spindle structure is formed in the nanofibers.

Furthermore, when the loading content is further increased, poor dispersity of POP NPs is observed, resulting in the aggregation of POP NPs and the formation of non‐uniform nanofibers. For instance, Yan et al.^[^ ^124^ ^]^ synthesized COF‐SCU1 through a Schiff base reaction involving triformyl chloride and p‐phenylenediamine as monomers at room temperature. They then prepared PAN@COF‐SCU1 nanofibers with varying loading amounts of COF‐SCU1 in the range from 10% to 30%, using a co‐electrospinning method. As the loading content increased further, more beads appeared in the resulting composite nanofibers. Zhang et al.^[^ ^126^ ^]^ reported a CUR@COF/PCL nanofibrous membrane containing curcumin‐loaded COF (CUR@COF), characterized by spindle‐shaped dark regions at a CUR@COF content of 5 wt.%. These dark regions indicated the presence of CUR@COF nanoparticles enclosed by the electrospun nanofibers. The addition of CUR@COF nanoparticles enhanced the elastic modulus and tensile strength of the CUR@COF/PCL nanofibrous membrane. However, at a loading content of 10 wt.%, these desirable mechanical properties were compromised, possibly due to increased rigidity and the presence of defects resulting from nanoparticle aggregation.

In the cases where POP NPs can be effectively dispersed in the solvent, uniform POPs@Polymer NFCOMs can be achieved even at higher POPs content. For example, Zhu et al.^[^ ^127^ ^]^ dispersed cationic PAF (iPAF‐6) in DMF with PES as the polymer matrix to produce rough nanofibers with a mean diameter of 500 nm at a POPs content of 30%. To further enhance the dispersibility and loading capacity of POP NPs in the composite nanofibers, Zhi et al.^[^ ^75^ ^]^ introduced a template‐oriented electrospinning method for constructing polymer nanofibers. Subsequently, a secondary polymerization and carbonization step was carried out to obtain nitrogen‐doped porous carbon nanofibers. In this process, a Schiff‐base oligomer (SBO) prepared through pre‐polymerization was initially electrospun into the PAN nanofiber matrix, followed by secondary polymerization to yield the final NFCOMs. The as‐electrospun nanofibers displayed a well‐defined microstructure at a SBO:PAN ratio of 1:1 and were interwoven into a nanofiber mat (Figure 10c). Despite substantial efforts, it remains a challenge to create POPs@Polymer NFCOMs with both uniform diameters and desirable functionalities using the direct blend electrospinning technique.

#### 2.2.2 In Situ Polymerization on Surface of Electrospun Nanofibers

For POPs synthesized under mild conditions, it is possible to create composite nanofibers decorated with POPs (Polymer@POPs) through in situ polymerization of POPs directly on the surface of electrospun nanofibers. The thickness of the POPs coating on the nanofiber surface can be fine‐tuned by adjusting parameters such as polymerization time and monomer concentration.^[^ ^129^ ^]^ To execute this method effectively, a polymer matrix with robust mechanical strength and chemical stability, especially under the solvent and temperature conditions, is required for POPs synthesis. PAN is a frequently selected nanofibrous polymer matrix for hosting POPs due to its exceptional thermal stability, solvent resistance, and excellent spinnability. For instance, Herath et al.^[^ ^130^ ^]^ achieved the synthesis of hybrid guanidinium‐based ionic COF (PAN‐BT‐DG) via an in situ condensation reaction involving N, N'‐diaminoguanidine (DG) monohydrochloride and benzene‐1,3,5‐tricarbaldehyde (BT) on PAN nanofibers. The resulting PAN‐BT‐DG nanofibers exhibited increased surface roughness and their average diameter expanded from 287 to 440 nm.

**Figure 11:**
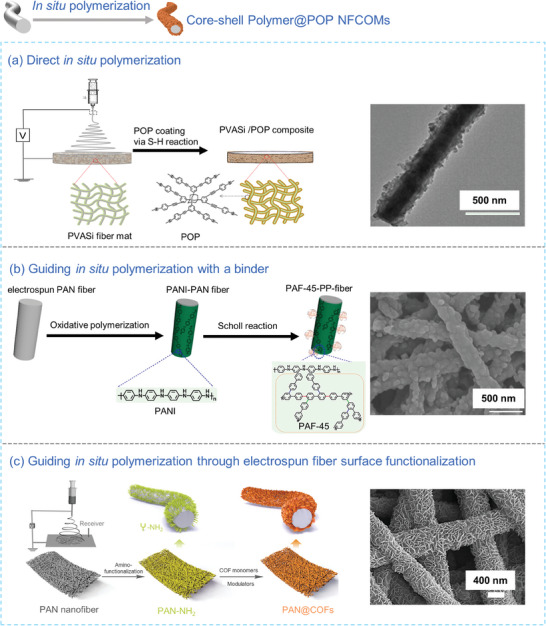
a) Synthetic scheme and SEM image of core‐shell POP NFCOMs by direct in situ polymerization method.^[^ ^133^ ^]^ Copyright 2019, Elsevier. b) Synthetic scheme and SEM image of core‐shell POP NFCOMs through the introduction of a binder to guide the in situ polymerization.^[^ ^134^ ^]^ Copyright 2019, American Chemical Society. c) Synthetic scheme and SEM image of core‐shell POP NFCOMs by functionalizing electrospun fiber surface to guide the in situ polymerization.^[^ ^139^ ^]^ Copyright 2023, Wiley‐VCH.

By making informed choices of monomers with well‐defined molecular structures and employing a nanofibrous polymer matrix, alongside adjustments in their noncovalent interactions like π‐π stacking and hydrogen bonds, it is possible to achieve a more uniform growth of POPs along the fibrous surface in Polymer@POPs NFCOMs compared to composite POPs@Polymer NFCOMs prepared via the direct blend electrospinning method. For example, Yue et al.^[133^^]^ presented a microporous polymer sponge reinforced by electrospun nanofibers through the in situ growth of CMP coatings on poly(vinyl alcohol) silica (PVASi) substrates using the Sonogashira‐Hagihara reaction (CMP/PVASi). CMP coatings exhibited uniform growth on the surface of the PVASi nanofibers, facilitated by hydrogen bonds between the primary amino groups in 2,5‐dibromoaniline (TEDB‐NH_2_) monomer and the ─OH groups in PVASi (**Figure** **11**a). Additionally, due to the crosslinking reaction between the ─OH groups in PVA and tetraethyl orthosilicate in the nanofiber matrix, the resulting composite nanofibers displayed excellent chemical and thermal stability. Zhu et al.^[^ ^134^ ^]^ initiated the process by coating polyaniline (PANI) on the electrospun PAN fibrous membrane, which acted as the aromatic seed layer to establish π‐π interactions and hydrogen bonding with PAF‐45, a PAF synthesized using biphenyl and Lewis acid (AlCl_3_) in a Scholl reaction. Subsequently, they conducted in situ grafting of PAF‐45 to obtain PAF‐45‐PP fibers (Figure 11b). This method uniformly distributed PAF nanoparticles on the fiber surface through noncovalent interactions. In another example, Tian et al.^[^ ^131^ ^]^ synthesized a PAN@COFs nanofibrous membrane by in situ polymerization of COFs nanospheres onto PAN nanofibrous membranes at room temperature. The growth of COFs onto the PAN nanofibers was facilitated by the presence of abundant proton acceptor groups (C≡N) in PAN and numerous proton donor groups (‐NH_2_, ‐CHO) in COFs, forming hydrogen bonds between them. It is important to note that the relatively weak noncovalent interactions impose limitations on the loading capacity of POPs in POPs@Polymer NFCOMs. These interactions also restrict the SSA and porosity of POPs@Polymer NFCOMs.

By introducing suitable functional groups to the electrospun nanofibers that can serve as nucleation sites, it becomes possible to create nanofibers adorned with uniform crystalline COFs. For instance, Thomas et al.^[^ ^139^ ^]^ engineered flexible COF nanofiber membranes by employing an in situ polymerization method for COFs on PAN nanofibers, following RPT approach. In this strategy, PAN nanofibers were modified with amino groups, which played a pivotal role as nucleation sites for the vertical growth of well‐aligned crystalline COF nanoplates, resulting in a highly porous structure. This approach readily grows crystalline COFs by in situ polymerization on electrospun nanofibers (Figure 11c).

#### 2.2.3 POP NFs Mat with Substrate‐Removal Method

**Figure 12:**
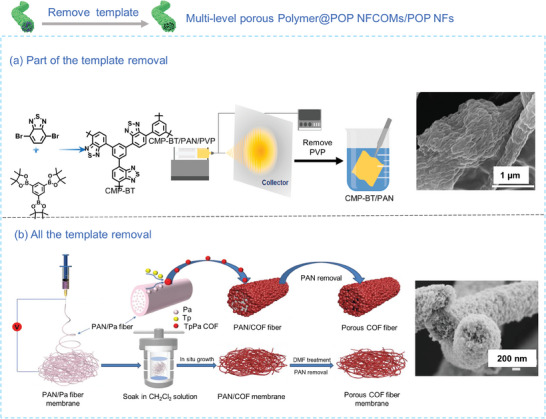
a) Synthetic scheme and SEM image of multi‐level porous NFCOMs by removing one of the component polymers in the template.^[^ ^128^ ^]^ Copyright 2020, Elsevier. b) Synthetic scheme and SEM image of multi‐level porous POP NFs by removing all the polymer template.^[^ ^71^ ^]^ Copyright 2021, Wiley‐VCH.

To expose more POP NPs that might be enclosed within the NFCOMs, POPs@Polymer NFCOMs with multilevel porosity have been developed. Lee et al.^[^ ^128^ ^]^ employed two distinct polymer matrices, PVP and PAN, to create benzothiadiazole‐based CMP (CMP‐BT)@PVP and PAN (P‐PAN) nanofibers with multilevel porosity. This was achieved by selectively removing only PVP and leaving only PAN as template (**Figure** **12**a). Generally, the mechanical properties of POP NFs mats obtained through this method tend to be relatively poor, and the final morphology typically comprises hollow nanofibrous structures. For instance, Chang et al.^[^ ^108^ ^]^ fabricated hollow nanofibrous CMP membranes by eliminating the PVP templates within the PVP@CMP electrospun membranes. The PVP@CMP fibrous membrane was initially created through the in situ polymerization of CMP on the electrospun PVP nanofibers, leading to an increase in the mean fiber diameter from 200 to 300 nm. After removing PVP matrix, the tensile strength of the CMP reached 3 MPa, and the resulting hollow tubular fibrous structure had an average wall thickness of ≈50 nm. Agarwal et al.^[^ ^71^ ^]^ utilized PAN nanofibers as sacrificial templates to produce self‐standing pure COF nanofibrous membranes. In contrast to the typical in situ polymerization of COF on PAN membranes, PAN and *p*‐phenylenediamine were blended in a mass ratio of 1:1 and electrospun into nanofibers (PAN/Pa‐100). Subsequently, composite nanofibers (PAN/COF100) was obtained by reacting with Tp in a Schiff base reaction. The removal of PAN resulted in COF100 nanofibers in a core‐shell structure (Figure 12b). Despite the reduction in mechanical strength of pure COF from 10.2 to 0.33 MPa due to the removal of the supporting matrix, the toughness of pure COF nanofibers was durable and remained unchanged even after subjecting them to 10 000 cyclic bending tests.

### 2.3 Preparation of POPs‐Derived Carbon Nanofibers

1D nanofibrous carbon materials have gained significant attention in various applications such as supercapacitors, microwave absorption, and pollutant removal. This is attributed to their enhanced electric properties, high SSA, lightweight nature, exceptional mechanical strength, and excellent chemical stability.^[^ ^54^, ^117^, ^140^, ^141^, ^142^ ^]^ Recently, there has been a growing interest in the design and fabrication of POPs‐derived nanoporous carbon nanofibers (PNCNFs). These PNCNFs are intriguing because of their distinctive hierarchical porous structures and the presence of heteroatom dopants.^[^ ^140^ ^]^ To achieve PNCNFs, HCPs have been widely employed as precursors for PNCNFs. HCPs are favored for their cost‐effectiveness, ease of preparation, and the distinctive 3D interconnection of microporous structures. A range of PNCNFs can be obtained through either the template‐free carbonization or template carbonization methods.

In the former, the synthesis of PNCNFs typically involves the carbonization of POP NFs without the use of a template. For instance, Xia et al.^[^ ^143^ ^]^ described the fabrication of crimped 1D fibrous LNU‐8 polymer through using 1,4‐dibromophthalene and 1,4‐ethylbenzene as building blocks in a Sonogashira–Hagihara coupling reaction. The development of 1D oligomers expanding in the planar direction was influenced by branching chain reactions, ultimately resulting in the formation of the desirable 1D fibrous structure. Following thermal annealing at temperatures ranging from 700 to 900 °C, LNU‐8‐based PNCNFs exhibited a hierarchical porous structure with macropores of ≈150 nm, mesopores at ≈5.2 nm, and micropores at 0.5 and 1.3 nm. It is worth noting that the pyrolysis temperature has a significant impact on the SSA of the final carbon materials, as too high temperatures can collapse the pre‐existing porous structure.

In the latter, electrospun nanofibers are commonly used as templates to facilitate the growth of POPs, enabling the fabrication of PNCNFs through pyrolysis at elevated temperatures. For instance, Wu et al.^[^ ^135^ ^]^ employed electrospun polystyrene (PS) nanofibers as the building blocks to create nanofibrillar morphologies in both POPs and PNCNFs, following a stepwise crosslinking process and subsequent pyrolysis. In the stepwise crosslinking procedure, glacial acetic acid was employed as a poor solvent for PS, effectively slowing down the rate of the Friedel‐Crafts reaction. This approach yielded precursors with a uniformly low‐crosslinking structure and nanofibrillar morphology. Control over the pre‐crosslinking degree, super‐crosslinking degree, and carbonization duration could produce porous carbons exhibiting a SSA value of 1615 m^2^ g^−1^ and excellent porosity. Compared to the template‐free carbonization method, the use of electrospun nanofiber templates significantly contributed to the construction of the porous structure during carbonization.

## 3 Applications for Nanofibrous POPs and their Derivatives

### 3.1 Water Treatment

#### 3.1.1 Pollutant Adsorption in Wastewater

**Figure 13:**
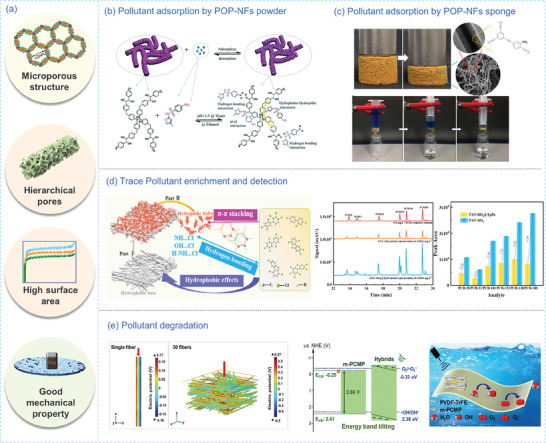
a) The benefits of employing nanofibrous POPs for wastewater treatment. b) Possible interaction mechanism of PPCPs adsorption on HPOP‐3 powder.^[^ ^66^ ^]^ Copyright 2022, Royal Society of Chemistry. c) Photo images showing the filtration process using a PVASi@TEDB‐NH_2_ sponge syringe filter.^[^ ^132^ ^]^ Copyright 2016, Elsevier. d) The enrichment mechanism and performance of PAN‐SiO_2_@TpPa on PCBs.^[^ ^145^ ^]^ Copyright 2023, Elsevier. e) The piezo‐photocatalytic mechanism of m‐PCMP/PVDF‐TrFE on removal of pollutants.^[^ ^146^ ^]^ Copyright 2022, Royal Society of Chemistry.

In contemporary aqueous systems, toxic organic pollutants, such as organic dyes, phenols, heavy metal ions, and emerging micropollutants like pharmaceutical and personal care products (PPCPs), constitute the primary source of water pollution. POPs exhibit exceptional properties, including a high SSA, tuneable pore size, enhanced chemical and thermal stability, and structural tunability (**Figure** **13**a). These merits make POPs promising for enriching and adsorbing pollutants from wastewater.

POP NFs, characterized by hierarchical porous structures and functional surface groups, exhibit high‐efficiency pollutant enrichment and capture. The introduction of hydroxyl (─OH) groups to the surface of POP NFs enhances their interaction with organic pollutants through non‐covalent forces. For instance, Ravi et al.^[^ ^66^ ^]^ synthesized three hypercrosslinked POPs with abundant ─OH groups via the Friedel–Crafts reaction to remove PPCPs. Among these, nanofibrous HPOP‐3, with a SSA of 552 m^2^ g^−1^, displayed the best adsorption performance due to easily accessible active sites on the pore surface. The adsorption of PPCPs onto the porous nanofibers followed the Langmuir model, suggesting uniform monolayer adsorption. HPOP‐3 exhibited adsorption capacities of 251.6, 331.9, and 380.8 mg g^−1^ for acetaminophen, sulfamethoxazole, and diclofenac sodium, respectively. The multiple interactions of the hydroxyl‐rich and aromatic HPOP‐3 with the pollutants, such as hydrophobic interactions, π‐π interactions, and hydrogen bonding, were identified as the driving force for adsorption (Figure 13b). Introducing functional groups like ─NH_2_, ─SO_3_H, and ─COOH enables electrostatic or chelating interactions to enhance the adsorption capacity of POP NFs toward metal ions.^[^ ^54^, ^130^, ^144^ ^]^ Ahmad et al.^[^ ^54^ ^]^ fabricated hollow tubular carbon nanofibers (HTnFs) by first cross‐polymerization of DCX and then carbonization. Subsequently, ─COOH and ‐SO_3_H groups were introduced onto HTnFs to adsorb uranium (VI) in water via electrostatic or chelating interactions between the functional surface of HTnFs and uranium (VI). The adsorption capacity of uranium (VI) reached as high as 1928.59 and 1827.57 mg g^−1^ at pH of 8.0 for ‐COOH and ─SO_3_H‐functionalized HTnFs, respectively.

The shaping of POP NFs into monolithic forms such as membranes or sponges with good mechanical properties endows the adsorbents with easy separation and excellent processability. The membranes possess microporous structures similar to the sponge. These microporous structures provide both membranes and sponges with increased reaction sites and enhanced selectivity. POPs‐based electrospun nanofibrous membranes are frequently used for wastewater purification due to their unique hierarchical porous structure. For example, Zhao et al.^[^ ^134^ ^]^ synthesized a PAF‐45‐PP electrospun fibrous membrane with a SSA of 262.4 m^2^ g^−1^ for the removal of PPCPs from water. The experimental findings indicated a good fit of the adsorption isotherm to the Langmuir model, revealing a maximum adsorption capacity of 613.50 mg g^−1^ for ibuprofen on PAF. The main driving forces for adsorption included π‐π interactions and hydrophobic interactions. In order to make active sites in POPs more accessible, Zhao et al.^[^ ^139^ ^]^ fabricated a PAN@COF nanofibrous membrane with vertical alignment of COF nanoplates growing on the nanofibrous surface. Such a membrane showed a nano‐micro hierarchical porous structure enabling high‐efficiency mass transport when removing ofloxacin, a typical antibiotic pollutant, with an adsorption capacity reaching 236 mg g^−1^.

In contrast to membranes, sponges with 3D network structures exhibiting unique compressibility can be used for fast adsorption and release of pollutants.^[^ ^132^, ^133^ ^]^ For instance, Kim et al.^[^ ^132^ ^]^ utilized a PVASi@TEDB‐NH_2_ sponge to treat methylene blue (MB)‐containing aqueous solutions. MB was immediately adsorbed by PVASi@TEDB‐NH_2_ via π‐π interaction. The adsorbed dye on PVASi@TEDB‐NH_2_ sponge could be regenerated by methanol washing, allowing the sponge to be reused. Additionally, due to its monolithic structure, it can be adopted as a syringe filter for continuous filtration of dyes in solution (Figure 13c).

#### 3.1.2 Trace Pollutant Enrichment in Wastewater

Incorporating POPs NPs with a large SSA and abundant functional groups into electrospun membrane has also been demonstrated effective for high‐efficiency enrichment and detection of trace micropollutants. For instance, Liu et al. in situ polymerized β‐ketoenamine‐linked COF on the NH_2_‐functionized PAN nanofibers to develop a COF‐modified nanofibrous membrane (PAN‐SiO_2_@TpPa) to efficiently concentrate and detect trace amounts of olychlorinated biphenyls in water.^[^ ^145^ ^]^ The membrane, characterized by numerous functional groups such as ─OH, ‐NH‐, and aromatic rings, facilitated various intermolecular interactions to enhance the enrichment of trace polychlorinated biphenyls (PCB) (Figure 13d). With excellent chemical and thermal stability, PAN‐SiO_2_@TpPa exhibited outstanding enrichment efficiency and robust reusability for trace PCB detection in diverse actual water bodies.

#### 3.1.3 Pollutant Degradation in Wastewater

The delocalization of π‐electrons in the conjugated skeleton of some POPs allows them to photocatalytically degrade organic pollutants. Engineering conjugated POPs into porous nanofibers not only makes the materials photocatalytically active but also preserves their structural integrity. For example, Lee et al.^[^ ^128^ ^]^ fabricated a CMP‐BT‐based composite nanofiber (CMP‐BT@P‐PAN), with PAN as supporting matrix and PVP as sacrificial porogen based on the electrospinning technology. CMP‐BT@P‐PAN displayed a degradation efficiency of 91% on rhodamine B (RhB) upon exposure to visible light, with electrons and superoxide radicals as the major contributor to degradation. Comparably, only 65% was obtained for nonporous nanofibers, underscoring the critical role of the porous structure. In order to promote the catalytic performance of POPs upon subjecting to mechanical stimuli, piezo‐photocatalysts can be designed to enhance electron transfer by introducing an electric field. For example, Meng et al.^[^ ^146^ ^]^ engineered piezo‐photocatalytic electrospun nanofiber membranes by incorporating photocatalytic pyridine‐based CMP (PCMP) into a solution containing piezoelectric poly(vinylidene fluoride‐co‐trifluoroethylene) (PVDF‐TrFE). The resulting composite nanofibrous membranes exhibited a degradation efficiency of 97% for RhB, highlighting the role of the electric potential of PVDF‐TrFE fibers in regulating the electric charges of PCMP (Figure 13e).

#### 3.1.4 Oil/Water Separation

**Figure 14:**
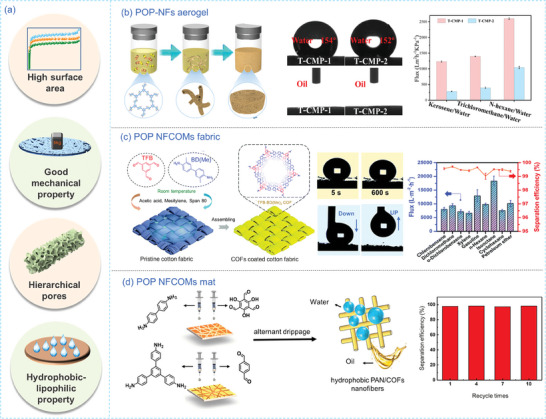
a) The benefits of employing nanofibrous POPs for oil‐water separation. b) Scheme showing the preparation process of POPs NFs aerogel, the contact angle test, and the separation flux of the T‐CMP nanotube filters.^[^ ^149^ ^]^ Copyright 2021, American Chemical Society. c) Scheme illustrating the fabrication process of POPs NFCOMs fabric, the contact angle test, and separation performance of TFB‐BD(Me)_2_‐COF‐coated cotton fabric.^[^ ^154^ ^]^ Copyright 2021, Royal Society of Chemistry. d) Scheme illustrating the preparation process of POPs NFCOMs mat, and its separation performance.^[^ ^129^ ^]^ Copyright 2022, American Chemical Society.

Nanofibrous POPs have demonstrated their effectiveness in separating oil/water mixtures. Their π‐conjugated skeletons provide them with unique hydrophobic‐lipophilic properties and robust chemical stability, making them excellent for adsorbing oil from oil/water mixtures. Furthermore, the hierarchical porous structures of nanofibrous POPs offer a large SSA and high mass transfer rates, enhancing their oil adsorption capacity (**Figure** **14**a). Additionally, the fabrication of nanofibrous POPs membranes ensures high permeability and separation efficiency in selective oil/water separation processes. To date, three types of superhydrophobic‐lipophilic or underwater superoleophobic nanofibrous POPs have been developed for oil/water separation, including template‐free POP NFs, POP NFCOMs, and POP NFs electrospun membranes.

Template‐free POP NFs offer advantages in terms of high adsorption capacity for organic matters because of their substantial SSA and hydrophobic structure when compared with their composite counterpart. For instance, Li et al.^[^ ^147^ ^]^ synthesized 1D CMP‐P nanotubes through a Sonogashira‐Hagihara cross‐coupling reaction using 1,3,5‐tribromobenzene and 1,4‐divinylbenzene as monomers. CMP‐P exhibited superhydrophobic properties and a large SSA, allowing it to absorb up to 12 times its own weight in organic solvents. CMPs/AAO composite membranes were then prepared using 1,3,5‐trivinylbenzene and 1,4‐dibromobenzene as monomers, with anodized aluminium oxide (AAO) serving as the inorganic template.^[^ ^148^ ^]^ The resulting CMPs/AAO composite membrane exhibited hydrophobic and lipophilic characteristics, effectively separating suspended oil from water with a separation efficiency of 99.4%. Moreover, another nanotube filters based on thienyl‐CMP (T‐CMP) were fabricated through a one‐pot method, achieving a separation efficiency of 96.5% and a flux of 1398 L m^–2^ h^–1^ kPa^–1^ for separating water‐in‐trichloromethane emulsions (Figure 14b).^[^ ^149^ ^]^ Despite achieving high‐performance oil/water separation, template‐free POP NFs face challenges related to their mechanical properties and flexibility, which require improvement for practical applications.

Loading POPs onto various fibrous substrates, such as stainless steel mesh,^[^ ^150^, ^151^ ^]^ polyester sponge^[^ ^152^, ^153^ ^]^ and cotton cloth,^[^ ^154^ ^]^ create POP NFCOMs with improved flexibility and mechanical properties compared to template‐free POP NFs. For instance, Jiao et al.^[^ ^155^ ^]^ prepared a superhydrophobic aminopyridine CMP and loaded it onto cotton fibers through physical impregnation to create a membrane device for oil/water separation. It's important to note that in the physical impregnation method, the distribution of POPs on the fibrous matrix is non‐uniform, and their interaction with the matrix is relatively weak. In contrast, in situ polymerization allows for the creation of a more uniform and tightly bound POPs coating. For instance, Dong et al.^[^ ^154^ ^]^ fabricated a 2D superhydrophobic COF‐coated cotton fabric by in situ polymerization (Figure 14c). This COF‐coated fabric exhibited a high surface area and maintained stable superhydrophobicity. It achieved a separation efficiency of 99% and a flux of 18000 L m^−2^ h^−1^ for the separation of water‐in‐isooctane emulsions. This superior performance can be ascribed to COFs’ relatively larger pore size (≈2 nm), allowing for oil compounds to efficiently pass through. It's worth noting that the efficiency and flux of oil/water separation in coated POP NFCOMs mats are influenced by the matrix's porosity, with fibrous matrices featuring hierarchical pores being particularly advantageous.^[^ ^119^ ^]^

Electrospinning technology is a valuable method for fabricating nanofibrous membranes for oil/water separation due to its simplicity in operation, cost‐effectiveness, and precise control over fibrous structures.^[^ ^142^, ^156^, ^157^ ^]^ For instance, Zhang et al.^[^ ^125^ ^]^ employed a blend electrospinning process to create bioinspired spindle‐knotted nanofibers. These nanofibers exhibited underoil superhydrophobicity and underwater superoleophobicity properties, enabling the separation of various oil/water mixtures. They achieved a separation efficiency exceeding 99.9% and a flux of 4229.29 L m^−2^ h^−1^. The spindle‐knotted structure, formed by adding COF, not only enhanced mechanical strength but also significantly increased separation efficiency. Zang et al.^[^ ^158^ ^]^ developed electrospun hydrophobic and oleophilic films by blending fluorine‐containing CMP (CMP‐F) with PI. The 3D network facilitated pathways for oil permeation, and the highly hydrophobic CMP‐F component repelled water from the membrane. As a result, the prepared membrane achieved a remarkable separation efficiency of 99.5% and a maximum flux of 13 500 L m^−2^ h^−1^ for hexane, maintaining these levels over 15 repeated uses. It also demonstrated a separation efficiency of up to 99.1% for water‐in‐toluene emulsion. To ensure uniform COF growth on the fibrous surface and maximize COF utilization, Chen et al.^[^ ^129^ ^]^ synthesized two different Schiff‐based COFs through in situ polymerization on electrospun PAN nanofibers. After hydrophobic modification with lauryl groups, the contact angle of the composite nanofiber membrane reached 167° against water, imparting excellent separation performance for various water‐in‐oil mixtures. Despite these advantages, it's essential that the polymer matrix used for spinning enhances chemical and/or solvent resistance. Furthermore, efforts are needed to enhance the recycling performance of oil/water separation (Figure 14d).

### 3.2 Gas Adsorption

Owing to their abundant micropores and tuneable functional groups, POPs can facilitate the adsorption of gaseous pollutants, e.g., iodine vapor, CO_2_, and fine particulate matters. POP NFs, with high porosity and well‐designed active sites, can deliver exceptional adsorption performance. For instance, El‐Kadri et al.^[^ ^110^ ^]^ synthesized azo‐functionalized pyrene‐based POP NFs (Azo‐Py), with thicknesses of ≈0.2 µm and various lengths achieved through an oxidative homocoupling reaction. The SSA and micropore volume of resulting Azo‐Py polymer were 1259 m^2^ g^−1^ and 0.80 cm^3^ g^−1^, respectively. It displayed impressive adsorption capacities for CO_2_, reaching 4.79 and 2.89 mmol g^−1^ at 273 and 298 K under 1 bar, respectively. Furthermore, its narrow pore size distribution of 1.06 nm endowed Azo‐Py with excellent adsorption selectivity, with CO_2_/CH_4_ (10.9) and CO_2_/N_2_ (55.1) ratios.

Nevertheless, handling bulky POPs particles can be quite challenging. An effective solution deals with incorporating POPs onto fibers, making them more convenient and practical to access. For instance, Li et al.^[^ ^57^ ^]^ designed a method to create COFs@cotton by grafting 2D‐COF onto cotton fibers through a Schiff base reaction involving two monomers: TAPB and terephthaldehyde. The abundant imine functional groups present in COFs@cotton gave it a strong affinity for iodine, resulting in an impressive capture capacity of up to 533.9 mg g^−1^. However, sodium iodate could harm the chemical structure of cellulose, leading to a low COF grafting rate and reduced iodine adsorption capacity. To overcome this, an alternative COF grafting method was explored. In this approach, cotton fibers were aminated via a silylation reaction involving (3‐aminopropyl)trimethoxysilane, followed by the in situ grafting of COFs.^[^ ^96^ ^]^ This modified process significantly improved the adsorption capacity of the resulting hybrid monolith for iodine, achieving an impressive 823.9 mg g^−1^.

Further structuring POPs into nanofibrous morphologies offers the advantage of easy integration into membranes or other adsorption devices, thereby enhancing their effectiveness in air pollutant separation. For instance, Meng et al.^[^ ^159^ ^]^ fabricated free‐standing hollow nanofiber membranes (PAF‐56P/PSF) by incorporating PAF‐56P nanoparticles into the PSF matrix. PAF‐56P, with its high SSA (553.4 m^2^ g^−1^) and numerous nitrogen species acting as active sites, demonstrated a strong affinity for CO_2_. The resulting membrane exhibited exceptional separation performance for CO_2_/N_2_, achieving a selectivity of up to 38.9. Bai et al.^[^ ^160^ ^]^ synthesized porous composite membranes (PCMs) through a one‐step click chemistry approach. These PCMs exhibited remarkable adsorption efficiency for PM10 and PM2.5, with removal rates of 99.89 ± 0.89% and 99.80 ± 1.58%, respectively, within 30 hours. The high capture performance of PCMs is ascribed to multiple functional groups present on their surface, such as unpaired electrons of S and C and aromatic rings, combined with their porous structure. Yan et al.^[^ ^136^ ^]^ developed flexible heterogeneous nanofibrous membranes (HNM) using a space‐constrained rigid‐flexible coupled hypercrosslinking method. HNM featured a mesoporous multi‐cavity network on its interior and a microporous network in the outer mesh. With a high SSA (579 m^2^ g^−1^) and hierarchically porous structure, HNM exhibited outstanding removal efficiency, capable of removing 2‐chloroethyl ethyl sulfide, a simulant for mustard gas in chemical warfare, at a rate of 498 mg g^−1^.

### 3.3 Energy Storage

**Figure 15:**
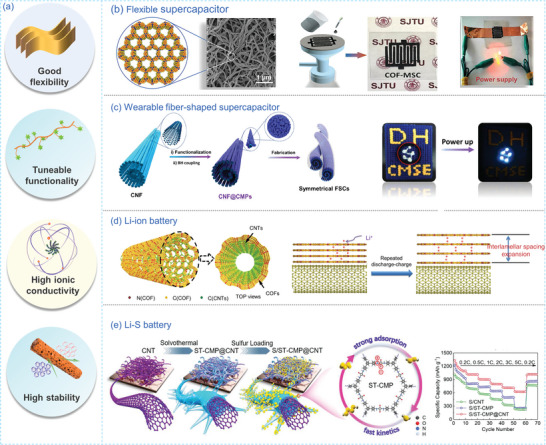
a) The benefits of employing nanofibrous POPs for energy storage. b) SEM images of *g*‐C_30_N_6_‐COF and photographs of COF‐MSC electrodes powering the red LED.^[^ ^107^ ^]^ Copyright 2020, Elsevier. c) Scheme showing the preparation of symmetrical FSCs and photographs showing powered LEDs and digital display by FSCs in series and parallel.^[^ ^98^ ^]^ Copyright 2020, American Chemical Society. d) Scheme showing COF@CNTs with a limited number of COF layers coating the outer surface of CNTs, and the facilitated transport and storage of lithium ions within expanded COF layers during the repeated discharging‐charging process.^[^ ^161^ ^]^ Copyright 2018, Springer Nature. e) Scheme showing the preparation process of S/ST‐CMP@CNT and rate performance of S/CNT, S/ST‐CMP, and S/ST‐CMP@CNT electrodes.^[^ ^167^ ^]^ Copyright 2021, Wiley‐VCH.

The designable porous structures and versatile functionalities of POPs materials make them promising for use in electrochemical energy storage (**Figure** **15**a), *e.g*., supercapacitors, lithium ion batteries, and emerging osmotic energy storage.^[^ ^98^, ^161^, ^162^ ^]^ Nevertheless, the limited processability and low electron conductivity of rigid, nanoparticulate POPs hinder their applicability. To address these challenges, the design and production of innovative nanofibrous materials based on POPs, including POP NFs, POP NFCOMs, and PNCNFs, hold potential as effective solutions.

Creating well‐defined nanofibrous materials not only retains the inherent properties of powderous POPs but also enhances their processability. For instance, Zhang et al.^[^ ^107^ ^]^ developed novel olefin‐linked COF nanofibrous thin‐film electrodes for flexible micro‐supercapacitors (MSC). These olefin‐linked COF nanofibers were synthesized by utilizing 2,4,6‐trimethyl‐1,3,5‐triazine as a key monomer that can be readily dispersed in polar organic solvents in a Knoevenagel condensation reaction. This improved processability facilitated their assembly with single‐walled carbon nanotubes to create binder‐free, free‐standing COF‐based interdigital MSC film electrodes (Figure 15b). Thanks to the presence of pores, numerous active sites, and efficient electron transfer channels, the resulting g‐C_30_N_6_‐COF‐MSC displayed an area capacitance of 44.3 mF cm^−2^ and demonstrated excellent cycling stability.

The electrochemical performance of materials is often closely linked to their electron conductivity. However, many POPs exhibit low electron conductivity. One solution to enhance electron conductivity is the in situ polymerization of POPs on electron‐conducting materials.^[^ ^91^, ^93^, ^163^, ^164^, ^165^ ^]^ For example, Xu et al.^[^ ^73^ ^]^ utilized carboxylated multi‐walled carbon nanotubes as an electrical substrate for the in situ growth of COF layers. They used 2,6‐diaminoanthraquinone (DAAQ) and TFP as monomers to create DAAQ‐TFP COF layers on the carbon surface. The resulting c‐CNT@COF nanofibers exhibited a core‐shell nanostructure, with a COF nanolayer as the outer coating and a c‐CNT skeleton as the inner core. This design harnessed strong π‐π interactions to facilitate electron transfer. A hybrid supercapacitor based on c‐CNT@COF demonstrated an area capacitance of 123.2 mF cm^−2^ and exhibited outstanding stability and flexibility for powering LED lights. Lyu et al.^[^ ^97^ ^]^ creatively grafted CMPs onto CNFs using a B‐H coupling reaction and produced macroscopic fibers based on CMPs for fiber‐shaped supercapacitors (FSCs) (Figure 15c). These fibers retained the necessary conductivity and flexibility of CNFs, while benefiting from the porosity and high redox properties of CMPs. As a result, a specific area capacitance of 398 mF cm^−2^ for the assembled two‐electrode symmetric FSCs were achieved. Even after enduring 10 000 bending cycles, the capacitance retained 84.5% of the initial value. Furthermore, FSCs in series or parallel configurations could be conveniently woven into wearable power sources for powering various wearable electronic devices.

In addition to improved electron conductivity, CNTs have demonstrated the ability to facilitate separation of few‐layered 2D COFs through π‐π interactions. This approach successfully addresses the challenge of buried interior redox‐active sites between layers in 2D COFs for lithium‐ion batteries (LIBs). For example, Wang et al. synthesized a few‐layer 2D imine‐based C = N coupled COF confined by CNTs (≈5 nm in thickness) (COF@CNTs). When used as an anode for LIBs, COF@CNTs displayed a capacity contribution of 1536 mAh g^−1^, maintaining 66% after 320–500 cycles. A 14‐lithium‐storage mechanism was proposed, wherein each C = N group accommodates one lithium ion, and an additional six lithium ions are stored individually on the benzene rings of a COF monomer. It is accomplished through the few‐layer lamellar structure of COF, facilitating electron conduction from CNT and gradual expansion of interlamellar spacing during repeated lithium insertion (Figure 15d).^[^ ^161^ ^]^

The excellent dispersion of some 1D materials facilitates the exposure of their active sites, thereby contributing to the enhancement of LIBs performance. To improve the accessibility of active sites on the electrode material to the electrolyte, a composite material with a 1D dendritic core‐shell structure (COF@CNT) was developed and implemented in the cathode of LIBs.^[^ ^166^ ^]^ The results indicate that the dendritic structure amplifies the density of redox‐active sites, thereby improving their utilization. The synergy between COF and highly conductive CNT imparted remarkable rate capability to COF@CNT, with an 82% retention maintained at 10 C, and excellent stability, maintaining 86% retention over 600 cycles at 5 C. The branched structure of porous polymers on CNT (ST‐CMP@CNT) can also act as nanotentacles, functioning as a high‐efficient electrocatalyst in Li‐S batteries.^[^ ^167^ ^]^ The copious polymer zwitterions, featuring both positively and negatively polarized atoms (N and O) in ST‐CMP, enable exceptionally high enriching and rapid catalytic conversion of LiPSs, showcasing commendable redox reaction kinetics. The initial specific capacity of resulting S/ST‐CMP@CNT composite electrodes was around 1307 mAh g^−1^ at 0.2 C, and the capacity maintained around 76.5% over 500 cycles at 1 C (Figure 15e).

In addition to POP NFs, PNCNFs demonstrate excellent conductivity along with high SSA, which can reach up to 1000 m^2^ g^−1^, making them fairly suitable for energy storage applications. For instance, Jiang et al.^[^ ^117^ ^]^ investigated PNCNFs derived from HCP nanotubes as electrode materials for supercapacitors. Their impressive characteristics include a high SSA of up to 921 m^2^ g^−1^, abundant nanopores, and uniform 1D channels, which accelerate ion diffusion within the electrode. In a symmetric two‐electrode configuration, these PNCNFs demonstrated remarkable cycling stability by retaining 93.6% of the initial capacitance after 5000 cycles. Furthermore, they displayed excellent rate performance, preserving 76.2% at 30 A g^−1^. The hierarchical pores in the PNCNFs provide efficient pathways for rapid ion transport, making them promising candidates for LIBs. For example, Zhang et al.^[^ ^100^ ^]^ employed MTCFs as anode materials for LIBs. The tubular nanostructure of MTCFs boasts multi‐level pore features and a large SSA, which offers a multitude of active sites and shortened pathways for lithium ions and electrons. By incorporating a small quantity of Fe_3_O_4_ nanoparticles, MTCFs demonstrated a reversible capacity of 1011.7 mAh g^−1^ over 150 runs and displayed outstanding rate performance as an anode material for lithium‐ion batteries (LIBs).

### 3.4 Heterogeneous Catalysis

**Figure 16:**
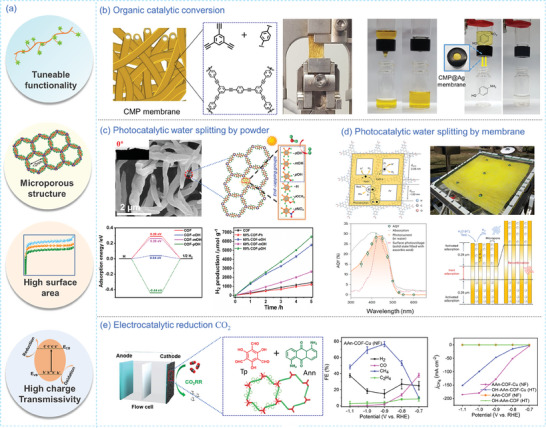
a) The list of benefits of employing nanofibrous POPs for heterogeneous catalysis. b) Synthetic route and performance on reduction of 4‐nitrophenol of CMP@Ag membrane.^[^ ^108^ ^]^ Copyright 2017, Springer Nature. c) Nanofibrous vinylene‐linked COFs powder with various end‐capping groups for photocatalytic hydrogen evolution.^[^ ^176^ ^]^ Copyright 2023, Elsevier. d) Chemical structure, device and performance of non‐woven/HOF‐H_4_TBAPy membrane for photocatalytic hydrogen evolution.^[^ ^177^ ^]^ Copyright 2023, Springer Nature. e) Schematic representation of Ann‐COF nanofibers for electrocatalytic CO_2_ reduction.^[^ ^104^ ^]^ Copyright 2021, Elsevier.

Engineering heterogeneous catalysis is significant for the large‐scale and selective formation of products, involving catalysts with different phases compared to the reactants and products. POPs have risen as advanced catalysts, thanks to their tuneable functionality, abundant micropores, large SSA and superior physicochemical stability (**Figure** **16**a).^[^ ^168^, ^169^, ^170^ ^]^ Nonetheless, there are challenges associated with dispersing POP NPs effectively in the reaction medium and isolating them from the reaction system.^[^ ^171^, ^172^ ^]^ The advent of 1D POPs addresses this issue. POP NFs, in particular, circumvent the drawback of aggregation of nanoparticles and ensure better exposure of active sites. It is widely believed that nanofibrous POPs can expedite charge transfer and enhance the catalytic process compared to nanofiber‐free aggregated nanoparticles.^[^ ^173^, ^174^, ^175^ ^]^ For example, Xia et al.^[^ ^116^ ^]^ conducted a performance comparison between non‐uniform pTTT‐Ben CMPs bulk particles and CMP nanofibers in the photocatalytic oxidation of 5‐hydroxymethylfurfural (HMF) and the hydrogenation of maleic acid (MA). The CMP nanofibers exhibited higher photocatalytic rates expectedly than CMPs nanoparticles. In another study, Bhaumik et al. synthesized nanofibrous polytriphenylamine (PPTPA‐1) through a one‐pot oxidative polymerization process, followed by sulfonation to create SPPTPA‐1 with fucntinal groups.^[^ ^101^ ^]^ Thanks to its high surface acidity, SPPTPA‐1 nanofibers efficiently converted sugar to HMF, achieving a conversion efficiency of 94.6% in merely 20 min. Furthermore, there was no significant loss of catalytic activity after 4 consecutive cycles.

POP NFs membranes have been developed to enhance the manageability of catalysts and address leaching issues. For instance, Chang et al. created a PVP@CMP electrospun hollow tubular membrane with CMP serving as the structural material. This was achieved through in situ polymerization in combination with a solvent extraction method.^[^ ^108^ ^]^ Owing to its high SSA, hierarchical pores and distinctive hollow tubular configuration, the subsequently prepared CMP@Ag polymer membrane with excellent stretchability facilitated the easy penetration of reactants through the CMP layer. This catalytic membrane significantly improved the performance in the catalytic reduction of 4‐nitrophenol and allowed for rapid regeneration. No weight loss was observed during consecutive catalytic cycles, and the attainment of complete conversion through gravity flow further underscored its user‐friendliness (Figure 16b).

The design and creation of appropriate photocatalysts are essential for achieving high rates of photocatalytic hydrogen production. Intriguingly, the fibrous morphology allows for the convenient handling of thin films as photoelectrodes without the need for additives. In comparison to photoelectrodes of other morphologies, nanofibrous COFs demonstrate significantly higher photocurrents and faster charge transfer rates. For instance, Zhang et al.^[^ ^106^ ^]^ synthesized various olefin‐linked COFs with well‐defined nanofibrillar morphologies using 1,3,5‐triazine as the core through Knoevenagel condensation. The resulting nanofibrous COFs exhibited a commendable photocatalytic hydrogen evolution activity of ≈290 µmol g^−1^ h^−1^, and there was no observable decline in hydrogen production activity after 4 cycles. The accelerated charge transport, facilitated by their coplanarity, is a possible reason for this boosted activity. Yang et al.^[^ ^176^ ^]^ further fine‐tuned the size and hydrophilicity of nanofibrous COFs by introducing various end‐capping groups at different positions in the nanofibrous vinylene‐linked COF. More importantly, this strategy effectively narrowed the energy barrier for H_2_ evolution. The optimized COF showed a 4.5‐fold improvement in hydrogen evolution activity under visible light compared to the unmodified olefin‐linked COF. Furthermore, it maintained good stability after long‐term cycling. (Figure 16c). More recently, Zhu et al.^[^ ^177^ ^]^ presented a flexible solar hydrogen evolution system by applying multiple drop‐coatings of the hydrogen‐bonded organic framework, 1,3,6,8‐tetrakis(p‐benzoic acid)pyrene (HOF‐H_4_TBAPy), onto a cellulose‐based nonwoven fiber. Due to the shortened transport path of excitons confined by micropores, the panel reactor exhibited a hydrogen generation rate exceeding 1 mol m^−^^2^ per day, with an apparent quantum yield exceeds 25% at 400 to 450 nm, and the high photoactivity can endure for 30 individual cycles, offering a viable strategy for large‐scale hydrogen production (Figure 16d).

Moreover, POP NFs demonstrate excellent electrocatalytic properties. In 2021, Lan et al.^[^ ^104^ ^]^ based on a self‐template mechanism, synthesized functionalized nanofibrous COFs (AAn‐COF) through a Schiff base condensation reaction. After modification by transition metals, AAn‐COF can highly fficient electrocatalytic reduce CO_2_ to CH_4_, as attributed to their larger electrochemical active surface area and exceptional charge transfer capability. Specifically, the AAn‐COF‐Cu nanofibers exhibited outstanding faradaic‐efficiency for CH_4_ (FE_CH4_) of 77% (128.1 mA cm^−2^, 0.9 V) in a flow cell, marking the highest reported FE_CH4_ among crystalline COFs. A durability test conducted at −0.9 V showed that the decay of FE_CH4_ for AAn‐COF‐Cu nanofibers was negligible over a 250‐minute period, indicating its excellent electrochemical stability. This represents the inaugural instance of anthraquinone‐based nanofibrous COFs for efficient electrocatalytic CO_2_ reduction to CH_4_, paving the way for investigating morphology‐controlled COFs in this domain (Figure 16e).

### 3.5 Other Advanced Applications

#### 3.5.1 Microwave Absorption

**Figure 17:**
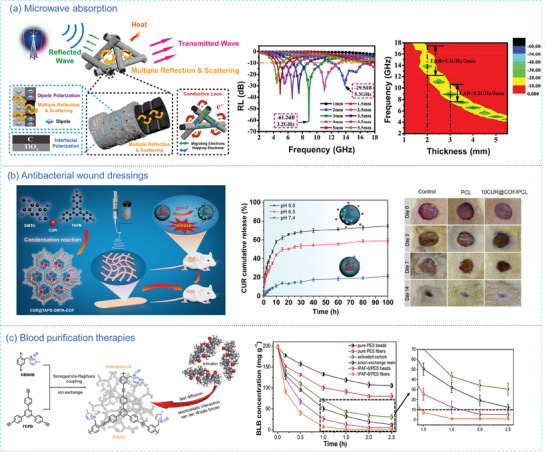
a) Schematic of microwave adsorption mechanism, RL curves and 2D contour curves of TCNFs@TiO_2_.^[^ ^140^ ^]^ Copyright 2020, American Chemical Society. b) Schematic of the preparation process of CUR@COF/PCL NMFs, drug release performance of CUR@COF/PCL NMFs and photographs of the wound dressing process.^[^ ^126^ ^]^ Copyright 2022, American Chemical Society. c) Scheme showing the preparation process of iPAF‐6, adsorption mechanism and performance of bilirubin with different adsorbents.^[^ ^127^ ^]^ Copyright 2020, Wiley‐VCH.

PNCNFs feature abundant interfaces, defects, high porosity, large SSA, and moderate and tuneable electrical conductivity, making them well‐suited for high‐performance microwave absorption applications. Typically, a combination of hybridization and carbonization methods is employed to tailor the microstructure and composition of PNCNFs to optimize their electromagnetic properties and energy conversion processes. For example, Zhu et al.^[^ ^140^ ^]^ designed a tubular carbon nanofiber@TiO_2_ (TCNFs@TiO_2_) hybrid structure (**Figure** **17**a). By adjusting the amount of TiO_2_ and the carbonization temperature, the dielectric properties of TCNFs@TiO_2_ can be fine‐tuned, enhancing its impedance matching behavior. Even with a 10 wt.% loading rate of TiO_2_, TCNFs@TiO_2_ exhibited an impressively effective absorption bandwidth of 5.3 GHz and a minimum reflection loss (RL) of −61.2 dB. This outstanding performance can be attributed to the favorable heterointerface in TCNFs‐TiO_2_, promoting interfacial polarization relaxation, and the hierarchical porous structure, which facilitates multiple reflections and scattering of microwaves.

#### 3.5.2 Biomedical Application

The tuneable pore size, morphology, surface chemistry and functionality of POPs provide the foundation for designing materials tailored for biomedical applications such as wound dressings and controlled drug release systems. For example, Landfester et al.^[^ ^178^ ^]^ employed a colloid‐electrospinning and crosslinking approach to embed benzo[c]−1,2,5‐oxadiazole‐based CMP nanoparticles within PVA hydrogel nanofiber membranes (PVA‐TBO) for antibacterial wound dressings. The exceptional mechanical properties confer easy‐removal and stability. The PVA‐TBO membrane achieved complete cell death for B. Subtilis and E. coli upon visible light irradiation for 60 min and 120 min, respectively, owing to the production of singlet oxygen (^1^O_2_) during the photocatalytic process with TBO. Zhang et al.^[^ ^126^ ^]^ designed a pH‐triggered drug release system by incorporating CUR@COF into PCL nanofiber membranes (CUR@COF/PCL NFMs) using the blend electrospinning technique. CUR@COF/PCL NFMs could release 29.8% of CUR at pH 5.0 and reach 74.6% after 100 h. The pH‐triggered drug release resulted from the protonation of imine groups and nitrogen species in C = N, which weakened the hydrophobic interaction between COF and CUR. Given the acidic extracellular microenvironment in tumor‐infiltrated and bacteria‐infected tissues, these delivery systems minimize drug loss in normal conditions and trigger rapid drug release under pathological conditions. In comparison to PCL, the fewest inflammatory cells were observed for 10CUR@COF/PCL, suggesting its favorable tissue healing properties (Figure 17b).

Another promising biomedical application for POP NFs is their use as bilirubin adsorbents in blood purification therapies. For example, Zhu et al.^[^ ^127^ ^]^ developed a high‐performance bilirubin removal membrane by electrospinning a composite dispersion of iPAF‐6 and PES. The iPAF‐6/PES nanofibrous membrane, enriched with multiple functional groups and high porosity, effectively reduces bilirubin levels to the normal concentration range (<10 mg g^−1^) within 1 h, outperforming commercial alternatives (Figure 17c). The primary driving force behind is proposed to be electrostatic and π‐π interactions between iPAF and bilirubin.

## 4 Conclusions and Perspectives

Recent research has centered on crafting POPs into nanofibrous structures, as reviewed here. Diverse synthetic methods, including direct bulk synthesis and electrospinning, have been employed to create nanofibrous POPs. The direct bulk synthesis method is time‐saving, efficient, and straightforward, yet it faces challenges in managing impurities (templates and small molecules) and achieving large‐scale production. While for electrospinning synthesis method, it is simple and efficient, but faces challenges in achieving uniformity and industrialization. It's important to note that the formation of nanofibrous POPs is influenced by several critical factors, often necessitating a combination of a specific fabrication approach and the carefully selected conditions involved to achieve well‐defined 1D structures. These developed nanofibrous POPs exhibit advantageous properties, such as high aspect ratios, substantial surface areas, hierarchical pores, large porosity, improved processability, and ease of use. Consequently, they hold great promise for diverse applications, ranging from environmental remediation to resource utilization, renewable energy generation and storage, and biomedical applications. In some cases, such as wastewater treatment, gas separation, biomedical uses, and heterogeneous catalysis, it is imperative to maintain the inherent functionalities of pristine POP NPs. In parallel, for applications predominantly related to energy storage, electronic conductivity is required when using them as electrodes. Abundant interfaces and defects are essential in the context of microwave absorption materials. Therefore, there is a rapid ongoing development of derivatives of nanofibrous POPs, including their conductive composites and carbon‐based nanofibers, to cater to these diverse needs.

Given the intricate nature of constructing nanofibrous POPs and their derivatives and their rapid advancement, we believe that further efforts in the field will be directed toward addressing the following issues: (1) The formation mechanisms of nanofibrous POPs in the bulk synthesis method remain incompletely understood, despite some proposed theories. (2) The large‐scale production of well‐defined nanofibrous POPs poses a challenge due to unclear assembly mechanisms and the relatively complex procedures involved in template methods and electrospinning technology. (3) The fabrication of electrospun nanofibers with highly exposed POPs active sites and robust mechanical properties is essential since POPs’ dispersibility in solutions is often poor. (4) The large‐scale manufacturing of nanofibrous POPs and their derivatives for engineering use is still in its infancy, akin to the early stages of nonwoven fiber processing. (5) Theoretical studies on the structure‐property relationship are lacking. Consequently, the development of more accessible and efficient materials synthesis and processing methods becomes crucial to realize cost‐effective, high‐performance nanofibrous POPs and their derivatives for broad practical use. (6) Attention is also required to investigate the technological impacts on the fabrication process of nanofibrous POPs and their derivatives and to discern the relationship between hierarchical porous nanofibrous structures and their performance.

In conclusion, functional nanofibrous POPs and their derivatives have displayed remarkable properties across various systems and potential applications. This review aims to sustain the ongoing research momentum, provide fresh inspiration, and offer novel insights for future work on nanofibrous POPs and their derivatives, addressing critical issues to facilitate their widespread utilization.

## Conflict of Interest

The authors declare no conflict of interest.

## Supporting information

Supporting Information
