# Supplementary material for: Nanofibrous Porous Organic Polymers and Their Derivatives: From Synthesis to Applications
Source: Adv Sci (Weinh). 2024 Mar 12;11(19):2400626. doi: 10.1002/advs.202400626 (PMC11109660; doi:10.1002/advs.202400626)
Supplement: Supplementary file 1 — Supporting Information [file ADVS-11-2400626-s001.pdf]

ADVANCED SCIENCE
Open Access

Supporting Information

for *Adv. Sci.*, DOI 10.1002/advs.202400626

Nanofibrous Porous Organic Polymers and Their Derivatives: From Synthesis to Applications

Chen Yang, Kexiang Wang, Wei Lyu, He Liu, Jiaqiang Li, Yue Wang, Ruyu Jiang, Jiayin Yuan* and Yaozu Liao**

Supporting Information

Nanofibrous Porous Organic Polymers and Their Derivatives: from Synthesis to Applications

Chen Yang[#], Kexiang Wang[#], Wei Lyu[*], He Liu, Jiaqiang Li, Yue Wang, Ruyu Jiang, Jiayin Yuan[*], Yaozu Liao[*]

Full name	Abbreviation
POPs nanofibers	POP NFs
POPs-based composite nanofibers	POP NFCOMs
POPs nanoparticles	POP NPs
POPs-derived nanoporous carbon nanofibers	PNCNFs
porous organic polymers	POPs
specific surface area	SSA
hypercrosslinked polymers	HCPs
polymers of intrinsic microporosity	PIMs
conjugated microporous polymers	CMPs
porous aromatic frameworks	PAFs
covalent organic frameworks	COFs
carbon nanofibers	CNFs
carbon nanotubes	CNTs
tetrabenzonaphthalene	TBN
Buchwald-Hartwing	B-H
polytriphenylamine	PTPA
polyimide	PI
polyacrylonitrile	PAN
1,3,5-triformylphloroglucinol	Tp
4,4′,4′′-(1,3,5- triazine-2,4,6-triyl) tribenzaldehyde	Tz
(R)-3 - amino - 4 - oxo - 2 - (pyrrolidin - 2 - yl) − 3,4 -	D-PDC

dihydroquinazoline‐7‐carbohydrazide	
(S)-3‐amino‐4‐oxo‐2‐(pyrrolidin‐2‐yl) – 3,4‐dihydroquinazoline‐7‐carbohydrazide	L-PDC
hexadecyltrimethylammonium bromide	CTAB
sodium dodecyl sulfate	SDS
triformylphloroglucinol phenylenediamine	TpPa
tubular polymer nanofibers	TPFs
magnetic TCFs	MTCFs
tubular carbon nanofibers	TCFs
formaldehyde dimethyl acetal	FAD
reversible polycondensation-termination	RPT
covalent organic nanotubes	CONTs
1,3,5-tris(4-aminophenyl)benzene	TAPB
tetraaminotriptycene	TAT
polycaprolactone	PCL
polyvinyl pyrrolidone	PVP
polyethersulfone	PES
polylactic acid	PLA
N,N-dimethylformamide	DMF
curcumin-loaded COF	CUR@COF
cationic PAF	iPAF-6
Schiff-base oligomer	SBO
benzothiadiazole-based CMP	CMP-BT
PVP and PAN	P-PAN
composite nanofibers decorated with POPs	Polymer@POPs
hybrid guanidinium-based ionic COFs	PAN-BT-DG
poly (vinyl alcohol) silica	PVASi
2,5-Dibromoaniline	TEDB-NH_2
polyaniline	PANI
polystyrene	PS
pharmaceutical and personal care products	PPCPs

hollow tubular nanofibers	HTnFs
α,α′-dichloro-p-xylene	DCX
methylene blue	MB
rhodamine B	RhB
poly(vinylidene fluoride-co-trifluoroethylene)	PVDF-TrFE
pyridine-based CMP	PCMP
aluminum oxide	AAO
nanotube filters based on thienyl-CMP	T-CMP
branched structure of porous polymers on CNT	ST-CMP@CNT
fluorine-containing CMP	CMP-F
azo-functionalized pyrene-based POP NFs	Azo-Py
porous composite membranes	PCMs
heterogeneous nanofibrous membranes	HNM
micro-supercapacitors	MSC
2,6-diaminoanthraquinone	DAAQ
fiber-shaped supercapacitors	FSCs
lithium-ion batteries	LIBs
5-hydroxymethylfurfural	HMF
nanofibrous polytriphenylamine	PPTPA-1
1,3,6,8-tetrakis(p-benzoic acid)pyrene	HOF-H4TBAPy
nanofibers	NF
tubular carbon nanofiber@TiO_2	TCNFs@TiO_2
reflection loss	RL
benzo[c]-1,2,5-oxadiazole based CMP nanoparticles embedded in PVA hydrogel nanofiber membranes	PVA-TBO
